# ON/OFF Photostimulation of Isatin Bipyridyl Hydrazones: Photochemical and Spectral Study

**DOI:** 10.3390/molecules24142668

**Published:** 2019-07-23

**Authors:** Róbert Šandrik, Pavol Tisovský, Klaudia Csicsai, Jana Donovalová, Martin Gáplovský, Róbert Sokolík, Juraj Filo, Anton Gáplovský

**Affiliations:** 1 Faculty of Natural Sciences, Institute of Chemistry, Comenius University, Ilkovičova 6, Mlynská dolina CH-2, SK-842 15 Bratislava, Slovakia; 2 Department of Pharmaceutical Chemistry, Faculty of Pharmacy, Comenius University, Odbojárov 10, SK-832 32 Bratislava, Slovakia

**Keywords:** on/off switches, isatin, hydrazones, UV-Vis spectroscopy

## Abstract

Four novel isatin hydrazones containing bipyridyl fragments were synthesized as potential ON/OFF switches. Hydrazone *Z*-isomers exhibit high thermal stability. The characteristic photochemical reaction for all studied hydrazones is the *Z*–*E* isomerization in CHCl_3_. After irradiation of hydrazones **1** and **2** in dimethylformamide (DMF), the photoreaction products are tautomers. When using light with the appropriate wavelength, the photo-tautomerization reaction is reversible. In these conditions, studied hydrazones have ON/OFF switch properties. In the case of hydrazones **1** and **2**, by alternating heat and light stimulation it is possible to control the isomerization process reversibly. In the presence of fluoride ions, NH hydrogen from the studied hydrazones is cleaved, and the corresponding anions are formed. The resulting anions of *Z*-isomers are changed to the corresponding *E*-isomer at room temperature.

## 1. Introduction

Molecular photoswitches are molecules that can be reversibly converted from one state (isomer) into another by light. Photochromic compounds are most often based on light-induced isomerizations around double bonds. Common switchable molecular motifs include azobenzenes [1], diarylethenes [2], spiropyrans [3], hemithioindigos [4], etc. The hydrazone structural motif is used in different fields of chemistry ranging from medicinal to supramolecular chemistry due to its facile synthesis, high stability, and unique structural properties [5,6]. An interesting feature of this molecules class is the photochemical, or chemical, *E*−*Z* isomerization around the imine-like C=N double bond, changing the configuration [7]. The reverse reaction can be both light-induced (P-type) or heat-induced (T-type), following either an out-of-plane rotation or a nitrogen inversion mechanism and consequently, the steric and electronic relation between the moieties on each side of the hydrazone core. Photoswitches now provide an invaluable tool for a large variety of applications in all fields of chemistry [8,9,10]. Although photochemical isomerization around the C=N double bond has been studied intensively in detail [11], it still attracts scientific attention because of the easy and reliable application of light as a stimulus in organic electronics (molecular switches) [5,6]. The resultant *E*/*Z* isomer ratio in the reaction mixture depends particularly on the thermodynamic stability of both isomers. In this synthesis, either the mixture of both *E*- and *Z*-isomers or the more energetically favorable isomer is obtained, and the second isomer is generally easily prepared by photochemical conversion [12]. 

**Scheme 1:**
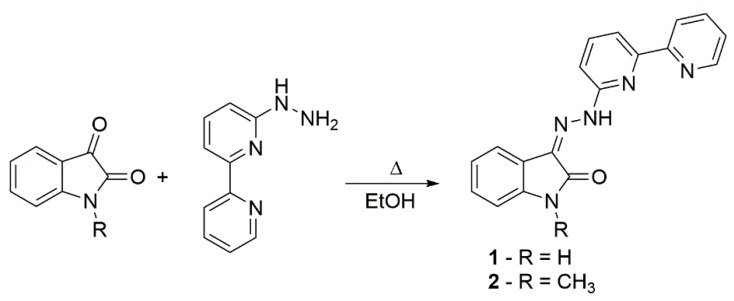
Synthesis of compounds **1** and **2**.

**Scheme 2:**
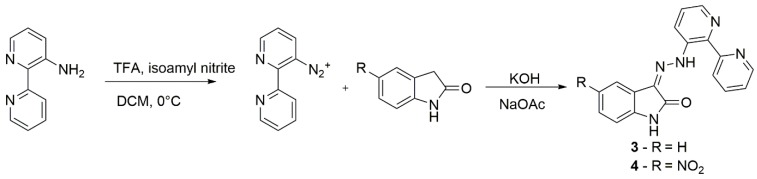
Synthesis of compounds **3** and **4**.

This paper investigates isatin bipyridyl hydrazones (Scheme 1 and Scheme 2), where NH hydrogen presence in the proposed new derivatives enables strong intramolecular hydrogen bonding. Isatin presence in the structure shifts their absorption to the vis-region of the electromagnetic spectrum. Detailed research was carried out to set the properties of these photoswitches as we plan to use these compounds as ON/OFF switches.

## 2. Results and Discussion

### 2.1. Synthesis

Compounds **1** and **2** were prepared by a condensation reaction of the corresponding isatin with 6-hydrazino-2,2’-bipyridine (Scheme 1). 

The corresponding products were isolated in high yields (74 and 76%). From the reaction mixture, we isolated desired products with the *Z*-isomer configuration. Since we could not prepare 3-hydrazino-2,2’-bipyridine, hydrazones **3** and **4** were prepared by reaction of diazonium salt from 2,2’-bipyridin-3-amine with corresponding oxindole (Scheme 2). In this case, yields were lower (52 and 47%). From the reaction mixture, pure *Z*-isomer of hydrazone **3** was isolated. In the case of hydrazone **4**, the *E*/*Z* ratio was 0.3.

### 2.2. UV-Vis and Fluorescence Spectra of Studied Hydrazones

**Figure 1:**
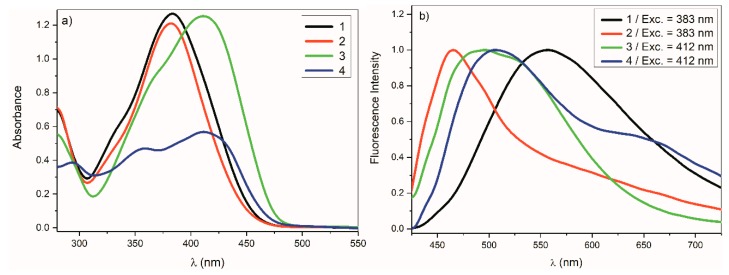
The studied hydrazones **1**–**4** (**a**) UV-Vis spectra (5 × 10^−5^ mol dm^−3^) and (**b**) normalized fluorescence spectra (5 × 10^−5^ mol dm^−3^) in dimethylformamide (DMF).

The studied compounds can be divided into two groups based on the bipyridyl fragment position in the hydrazone. The first group consists of compounds **1** and **2** with bipyridyl bound at the 6-position. The structure of the second group—compounds **3** and **4**, where the bipyridyl fragment is attached at the position 3—has been designed so that even the *E*-isomers are stabilized by intramolecular hydrogen bonding, like the aryl hydrazones *Z*-isomers [13,14]. The studied isatin arylhydrazones long-wavelength absorption maximum depends on their structure and is in the region from 370 nm to 420 nm (Figure 1). 

Compounds **3** and **4** have the long-wavelength absorption maximum batochromically shifted by approximately 28 nm compared to hydrazones **1** and **2**. Hydrogen bonding contributes to the hydrazone molecule planarity. This band split in the case of the hydrazone **4** is due to the NO_2_ group presence at the isatin 5-position. The NO_2_ group assists in the charge transfer through the isatin aromatic part [15]. Absorption maximum position dependence on solvent polarity shows little negative solvatochromism ranging from 4 nm to 8 nm (Table 1).

**Scheme 3:**
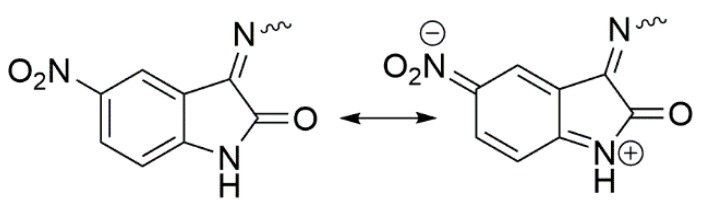
Charge transfer in the molecule of hydrazone **4**.

In the case of compounds **1**, **3**, and **4**, the fluorescence peak position in DMF compared to CHCl_3_ is batochromically shifted (Table 1). From the relatively large Stokes shift values in DMF (Table 1) it follows that charge transfer for hydrazones **1**, **2**, and **4** is large and approximately the same. A significantly smaller Stokes shift (50% reduction) was observed by compound **3**. We assume that this difference results from different charge transfer pathways in the studied compounds. For compounds **1** and **2** in DMF, formed tautomers (Supplementary Table S1) are more stable than hydrazones **3** and **4**. The nitro group at the position 5 on isatin of compound **4** reduces the charge transfer efficiency through the –NH–CO– fragment via the isatin 5-membered ring. In a high dielectric constant environment, the NO_2_ group mesomeric effect alters the charge distribution in the isatin fragment so the isatin amide nitrogen acquires a significant proportion of the sp^2^ character (Scheme 3):

The quantum-chemical calculations (M062X 6-31+g(dp), Supplementary Table S1) show that this charge transfer also occurs in a vacuum, especially in the *E*-isomer from hydrazone **4**. Chloroform stabilizes excited electron states significantly less than DMF. The lower stabilization is reflected at lower Stokes shifts in chloroform, but especially for the nitro derivative **4** (Table 1). Deactivation of the excited state by CT (charge-transfer) electron transfer significantly affects the fluorescence intensity and electron states lifetime, especially τ^1^. Fluorescence quantum yields of hydrazone **1**, **2**, **4** were <0.01. The fluorescence quantum yield of hydrazone **3** was 0.04 in DMF and 0.02 in CHCl_3_ (Table 1).

### 2.3. Photochemical and Thermal Reactivity of Hydrazones 

A characteristic photochemical reaction for hydrazones is geometric *Z*–*E* isomerization around C=N double bond [6,16]. Reversible *E*–Z isomerization was also observed for irradiation in all studied hydrazones **1**–**4** in non-polar solvents with low dielectric constants (CHCl_3_) (Supplementary Figures S2a,c, S3a,b, and S4). In mono-substituted isatin arylhydrazones, the *Z*-isomer is almost always more thermodynamically stable. By the reaction conditions, pure *Z*-isomers were prepared by the appropriate choice or by heat treatment in the solid phase. For compound **4**, from the reaction mixture, both isomers were obtained at a *Z*/*E* = 0.3 ratio. For the hydrazones **1**, **2**, and **3**, the heat-stimulated *Z*–*E* isomerization rate at room temperature was negligible. We did not observe any changes in UV-Vis spectra at 80 °C (6 h) in DMF. In the case of hydrazone **4**, thermally stimulated E–Z isomerization occurred in DMF (Supplementary Figure S1). The hydrazones **3** and **4** thermal *E*-Z isomerization rate constants are shown in Table 2. The hydrazones **1** and **2** *E*-isomers in DMF could not be prepared at concentrations that would allow their reliable quantitative evaluation by UV-Vis and NMR spectroscopy. In DMF hydrazones **1** and **2** the photochemistry is different from those of hydrazones **3** and **4**. The *Z*–*E* isomer ratio in the equilibrium reaction mixture after irradiation of the studied hydrazones in DMF (λ_irr_ = 370 nm) and CHCl_3_ (λ_irr_ = 405 nm) is in Table 2. 

**Figure 2:**
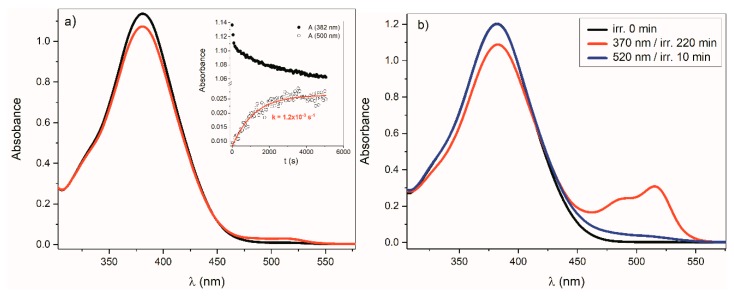
Hydrazone **2** (5 × 10^−5^ mol dm^−3^) UV-Vis spectra change: (**a**) heat-stimulated change (80 °C) (**b**) reversible light-stimulated change in DMF.

In the case of hydrazones **1** and **2** in DMF, the *Z*–*E* photoisomerization conversion is low (Table 2). From the UV-Vis spectra (Supplementary Figures S3c,d, S4b, and Figure 2b) it follows that during the irradiation at room temperature the absorption band intensity at 380 nm decreases and at the same time new multiple absorption bands in the range from 450 nm to 560 nm are formed. The same change in UV-Vis spectra of the *Z*-isomers was also observed at 80 °C in DMF (Figure 2a). Such behavior during photolysis was also observed in the case of other isatin arylhydrazones with strongly deactivated aryl [13,17]. 

**Scheme 4:**
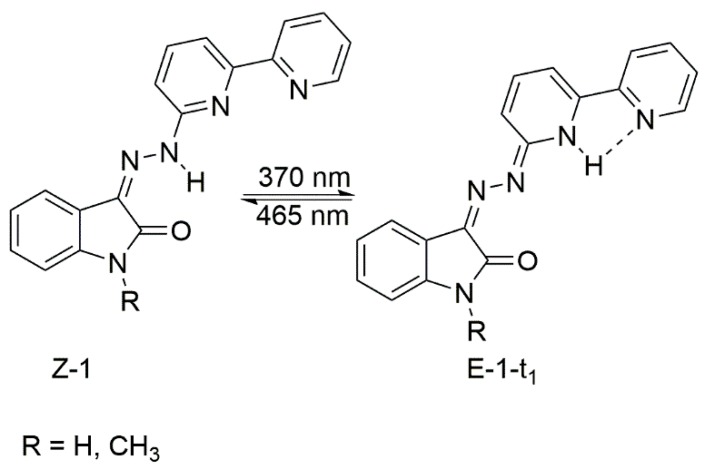
Light-initiated reversible tautomerization of hydrazones **1** and **2** in DMF.

**Figure 3:**
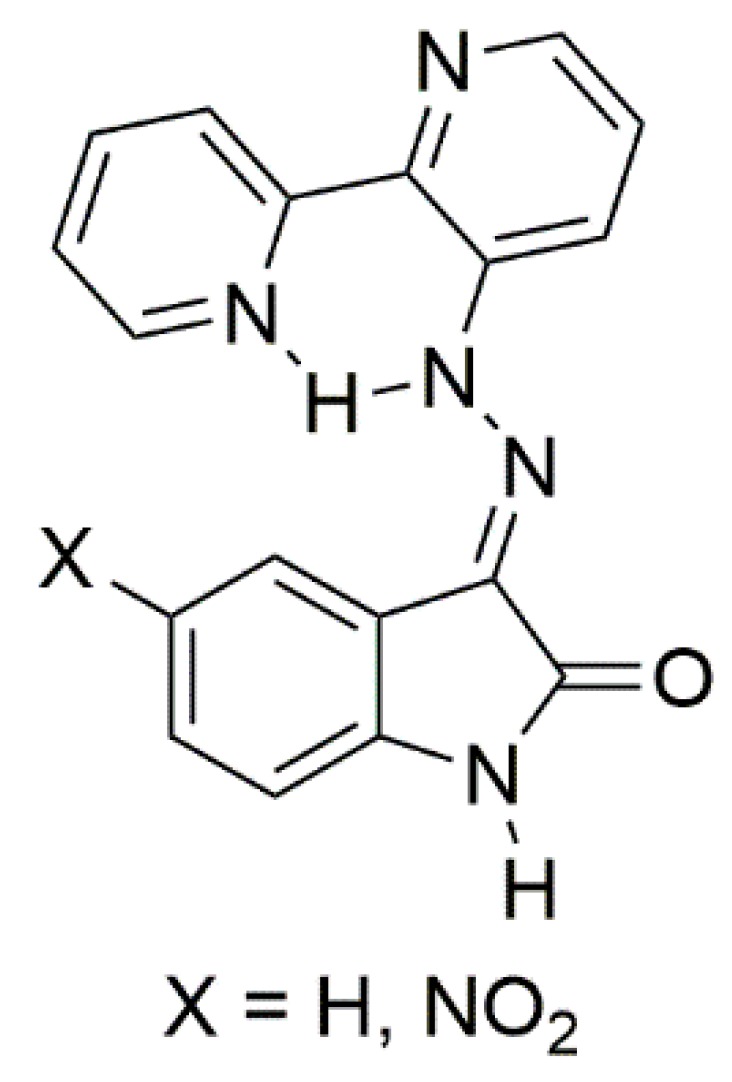
Stabilization of *E*-isomers from hydrazones **3** and **4** by an intramolecular hydrogen bond.

During the irradiation of hydrazones **1** and **2** in DMF, light-stimulated tautomerization occurs (Scheme 4):

From the hydrazo form, several tautomeric forms or conformers can be theoretically formed. The results of the quantum-chemical calculations confirm this. The thermodynamically most stable form is the (*E-1*-t*_1_*) form (Supplementary Table S1). The tautomerization process is reversible. Irradiation (λ = 465 nm) yields the hydrazoform *Z-1* (Scheme 4). Based on quantum-chemical calculations, we assume that the different photochemical reactivities of hydrazones **1** and **2** and hydrazones **3** and **4** are determined by the relative thermal stability of *E*-isomers and corresponding stability of tautomeric forms. Hydrazones **3** and **4** are geometric *E*-isomers stabilized by intramolecular hydrogen bonding (Figure 3).

**Figure 4:**
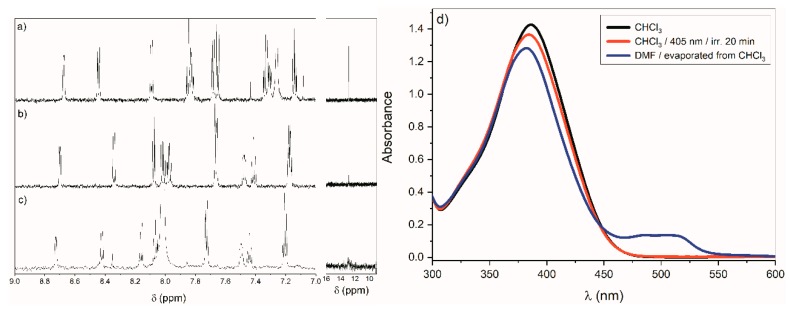
Hydrazone **2** tautomerization visualized by ^1^H-NMR: (**a**) 1 × 10^−3^ mol dm^−3^ in CDCl_3_; (**b**) 1 × 10^−5^ mol dm^−3^ in CDCl_3_ irradiated at 405 nm for 20 min; (**c**) solid from solution **b** in DMF-*d_7_*; and (**d**) UV-Vis spectra (5 × 10^−5^ mol dm^−3^) in DMF or CHCl_3_.

Thermodynamically, the hydrazone **3** *E-* and *Z*-isomers (*E-3* and Z-3) (Supplementary Table S1) are in DMF significantly more stable than the tautomeric forms (*Z-3-t_1_*); (*Z-3-t_2_*); (*E-3-t_1_*); (*E-3-t_2_*); and (*E-3-t_3_*). Therefore, during irradiation, *Z*–*E* isomerization occurs rather than tautomerization. In the case of hydrazones **1** and **2**, a similar stabilization of the *E*-isomer by intramolecular hydrogen bonding is not possible. The isomer molecules *E-1*, *Z-1-t_1_*, and the *E-1_-_t_1_* tautomer have almost the same Gibbs energy that differs from the most stable *Z-1* isomer in DMF by about 13 kJ/mol (Supplementary Table S1). Therefore, during irradiation, tautomerization occurs. Thermal tautomerization occurs in DMF only from the *E-1* isomer. Such a statement results from the following experimental findings: after irradiation (405 nm) of hydrazone **2** *Z*-isomer in CHCl_3_ a solid mixture of *Z*- and *E*-isomers was obtained after evaporation of the solvent. From NMR and UV-Vis spectra (Figure 4) it was evident that a tautomerization product was present in the solution upon dissolution of this mixture of isomers in DMF. *Z*-isomer does not provide such spectral changes in DMF. For the *Z-1* isomer, this absorption could be observed only after irradiation by light with a wavelength of 370 nm at temperatures at which *Z*–*E* isomerization occurs (Figure 4a–c). 

During this irradiation, the *E-1*-isomer is formed, which then rapidly tautomerizes. Table 2 shows the thermal tautomerization rate constants k_t_. Since only the *E*-isomer is thermally tautomerized and the tautomerization reaction rate is high, the thermal *Z*–*E* isomerization rate is the rate-determining step in this process. The measured rate constants of thermal tautomerization k_t_ characterize the hydrazones **1** and **2** thermal *Z*–*E* isomerization rate.

### 2.4. Studied Hydrazones N-Anions Thermal Reactivity

#### TBAF Titration—UV-Vis Spectra

**Figure 5:**
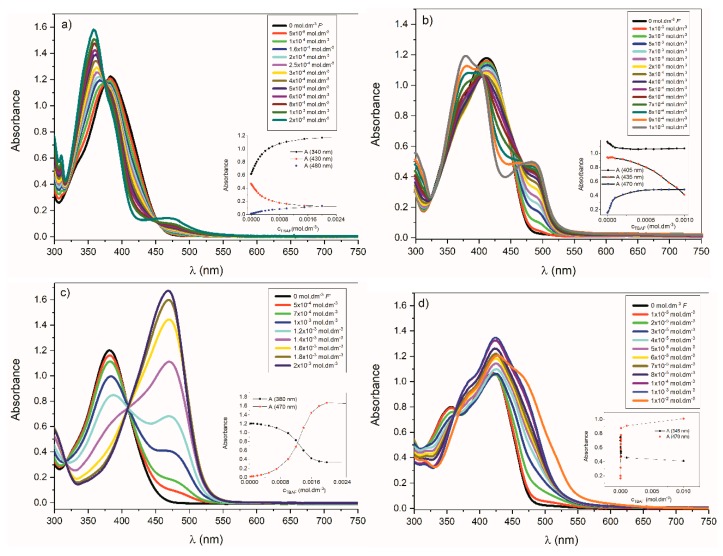
Titration of hydrazones with TBAF in DMF: (**a**) hydrazone **1** (5 × 10^−5^ mol dm^−3^); (**b**) hydrazone **3** (5 × 10^−5^ mol dm^−3^); (**c**) hydrazone **2** (5 × 10^−5^ mol dm^−3^); (**d**) hydrazone **4** (4 × 10^−4^ mol dm^−3^).

During titration of hydrazones **1**–**4**, like that observed for other isatin hydrazones [13,14,18,19] with basic F^−^ ions, we expected a new intense batochromically shifted band in the UV-Vis spectrum. But, by hydrazones **1** and **3** titrations in DMF with increasing tetrabutylammonium fluoride (TBAF) concentration, the starting hydrazone band intensity at 384 nm (413 nm for hydrazone **3**) decreased, and two new absorption bands were formed (Figure 5a,b). 

For hydrazone **1**, the new intense absorption band was hypsochromically shifted (maximum at 358 nm). The second low-intensity absorption band had a maximum at 475 nm. Hydrazone **3** had the maximum of the new intense absorption band at 378 nm and the low-intensity of the low-intensity absorption band at 486 nm (Figure 5a,b). A similar low-intensity absorption band corresponding to the isatin N-anion at 500 nm was also observed in the case of other isatin derivatives [15]. *N*-alkylated isatins did not have such an absorption band in the UV-Vis spectrum in the presence of F^−^ ions. In UV-Vis spectra, we did not observe isosbestic points during the TBAF titration of hydrazones **1** and **3**. During the titration, more equilibrium reactions were present (Figure 5a,b). The hydrazone **2** had the isatin NH hydrogen substituted by a methyl group. This NH hydrogen absence in the hydrazone molecule changed the titration course with the F^−^ ions (Figure 5c). At titration, only one intensive batochromically shifted band was formed in the UV-Vis spectrum of hydrazone **2** with a maximum at 470 nm. Based on these experiments, the hypsochromic shift of UV-Vis spectra of hydrazones **1** and **3** in the presence of F^−^ ions in DMF was due to the formation of (*Z-1-A_1_i*) and (*Z-3-A_1_i*) anions. These anions were formed by cleavage of NH hydrogen from the isatin fragment of hydrazones **1** and **3**. For the studied hydrazones, measured pK_a_ values (Table 3) support such a conclusion [20]. 

In hydrazones **1** and **4** both signals disappear immediately after the D_2_O addition. It is not possible to decide which of the NH hydrogen was changing faster. The hydrazone **4** titration by fluoride ions was complicated by the *E*- and *Z*-isomers presence in the reaction medium. 

Supplementary Figure S6 shows that immediately after the TBAF addition to the hydrazone **4** solution in DMF, a new batochromically shifted absorption band (473 nm) was formed. After reaching thermal equilibrium (25 °C and c_TBAF_ = 5 × 10^−3^ mol dm^−3^) the band maximum changes from 473 nm to 426 nm. From the chromatograms (Supplementary Figure S6) it is evident that after the TBAF addition to hydrazone **4** solution, there was also a change in the isomer proportions in the solution. 

**Scheme 5:**
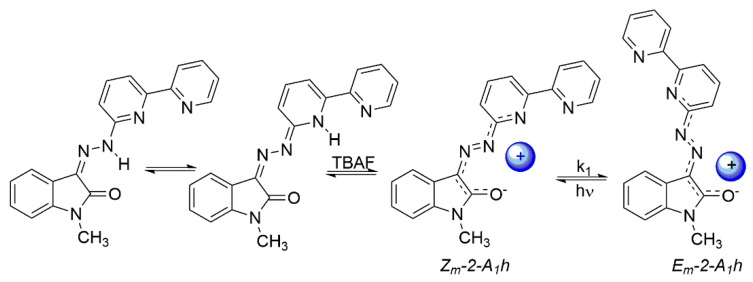
TBAF effect on hydrazone **2**.

After the TBAF addition to the hydrazones **1** and **3** *Z*-isomers, the *Z*-isomer N-anions (*Z_-_1-A**_1_**i*) and (*Z-3-A**_1_**i*) were formed by cleavage of the NH hydrogen from the isatin. In hydrazone **2**, the NH hydrogen was substituted by a methyl group and therefore during the titration, immediately after the TBAF addition, the *Z*-isomer N-anion from hydrazone (*Z_m_-2-A_1_h*) was formed (Scheme 5).

This NH hydrogen cleavage vanished the intramolecular hydrogen bond in the hydrazone molecule. The hydrogen bond disappearance and the Coulombic interaction of the negative charge located on the hydrazone nitrogen and the carbonyl oxygen created conditions for the easy thermal *Z*–*E* (*Z_m_-2-A_1_h*) anion isomerization.

**Figure 6:**
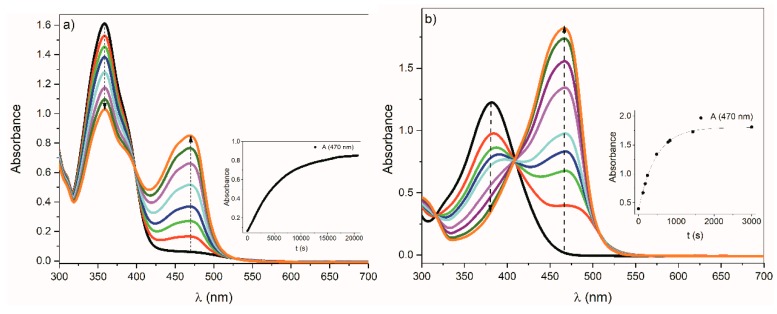
Anion thermal *Z*–*E* isomerization kinetics in DMF at 25 °C: (**a**) anion *Z-1-A_1_h* (5 × 10^−5^ mol dm^−3^) + 1 × 10^−3^ mol dm^−3^ TBAF; (**b**) anion *Z-2-A_1_h* (5 × 10^−5^ mol dm^−3^) + 1 × 10^−3^ mol dm^−3^ TBAF.

**Figure 7:**
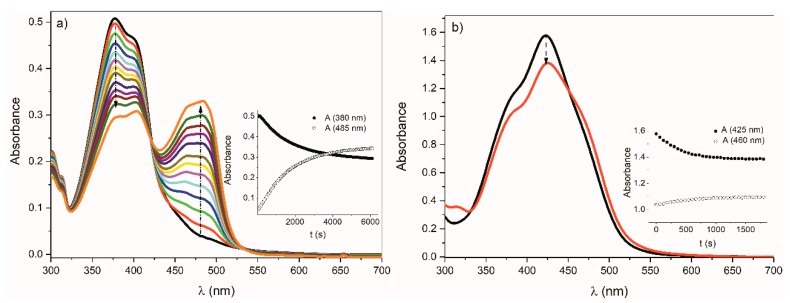
Anion thermal Z–E isomerization kinetics in DMF at 25 °C: (**a**); anion *Z-3-A_1_h* (5 × 10^−5^ mol dm^−3^) and 1 × 10^−3^ mol dm^−3^ TBAF; (**b**) anion *Z-4-A_1_h* (5 × 10^−5^ mol dm^−3^) + 1 × 10^−3^ mol dm^−3^ TBAF.

In the case of the studied hydrazones, during titration with fluoride ions, this process was reflected in the UV-Vis spectrum, as a gradual increase in the long-wavelength absorption band intensity and its low hypsochromic shift (Figure 6 and Figure 7). This long-wavelength absorption band in the studied hydrazones was composed of overlapping absorption bands of anions formed during titration. The rate constants of *Z*–*E* isomerizations of the anions from studied hydrazones are shown in Table 3. From the rate constants (Table 3), it is evident that the (*Z_m_-2-A_1_h*)–(*E_m_-2-A_1_h*) or (*Z-y-A_1_h*)–(*E-y-A_1_h*) isomerization of anions was faster than (*E_m_-2-A_1_h*)–(*Z_m_-2-A_1_h*) or (*E-y-A_1_h*)–(*Z-y-A_1_h*) isomerization, (y = 1,3,4). 

The *Z*–*E* and *E*–*Z* isomerization rate constants of anions (*Z-4-A_1_h*) and (*E-4-A_1_h*) are comparable. 

The rate constants k _E-Z_ of the anion isomerization for the anion (*Z-1-A_1_h*) were about 30 times and for the anion (*Z-3-A_1_h*) about 20 times smaller than k_E-Z_ of the anion from hydrazone **4** (*Z-4-A_1_h*). (Table 3). The rate constants of the hydrazone **3** *E*–*Z* isomerization and the rate constant of the corresponding anion (*E-3-A_1_h*)–(*Z-3-A_1_h*) isomerization had approximately the same value (Table 2 and Table 3). This is because of the intramolecular hydrogen bonding contribution to the stabilization of hydrazone *E*- and *Z*-isomers, which was mostly eliminated by carrying out the isomerization at 80 °C. The transformation of the *Z*-3 and *E*-3 isomers was dependent on the F^−^ ion concentration. After addition of fluoride ions c < 1 × 10^−4^ mol dm^−3^ to the mixture of *E*-3 and *Z*-3 isomers in DMF the absorbance at 480 nm in UV-Vis spectra increased. In the case of the *Z*-isomer, at these concentrations, such a change in UV-Vis spectra was not observed (Supplementary Figure S7). The increase in absorbance is due to the *E*-3-isomer presence in solution. The *E*-3-isomer is more reactive, more sensitive to the presence of F^−^ ions than the *Z*-isomer. Similarly, the *Z*-isomer N-anion, (*Z-3-A_1_h*) and *E*-isomer N-anion, (*E-3-A_1_h*) are under these conditions thermally unstable. However, in UV-Vis spectra, we did not observe an increase in absorbance at 480 nm depending on the reaction time. The absorbance at 480 nm decreased with time while the absorbance at 410 nm increased.

**Figure 8:**
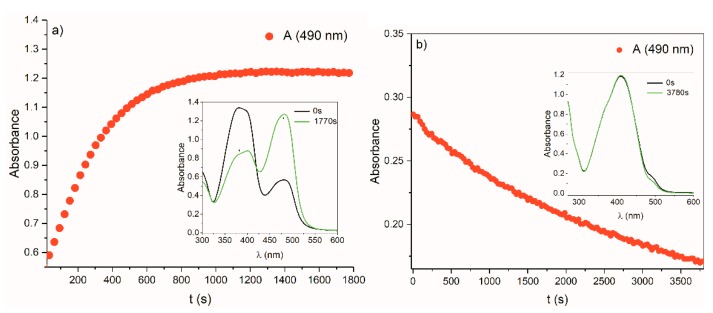
Hydrazone **3** reaction kinetics (5 × 10 mol dm^−3^) with TBAF in DMF at 50 °C: (**a**) 1 × 10^−2^ mol dm^−3^ TBAF; (**b**) 5 × 10^−4^M TBAF. Inset: hydrazone **3** UV-Vis spectrum change in dependence on time.

**Scheme 6:**
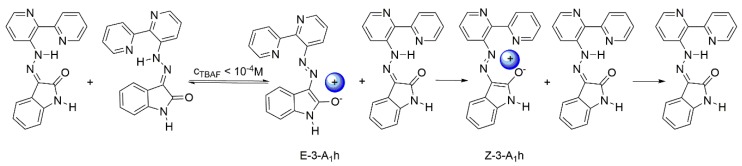
*E*- and *Z*-isomer transformation process at low TBAF concentration.

It is, therefore, a reversible process. The UV-Vis spectrum thus takes the form of a spectrum corresponding to the *Z*-isomer (Figure 8b). The entire transformation process of *E-* and *Z*-isomers at low TBAF concentration (<10^−4^ mol dm^−3^) is described in Scheme 6. 

**Scheme 7:**
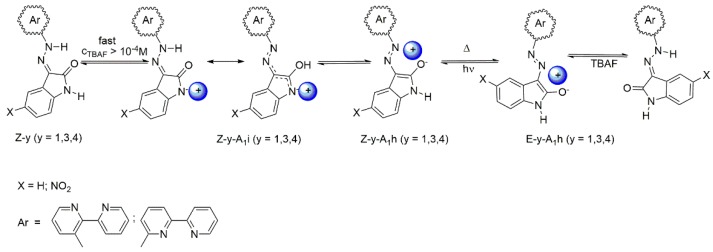
Kinetics of formation and transformation of anions from studied hydrazones.

There is no direct evidence of whether under these conditions N-anion (*Z-3-A_1_h*) is formed by the anion (*E-3-A_1_h*) transformation or by the direct cleavage of the NH hydrogen from the hydrazone fragment. It is not possible to decide clearly even based on the NH hydrogens H–D exchange (Supplementary Figure S8). However, the observed changes in UV-Vis spectra (decrease in absorbance at 480 nm with simultaneous reduction of absorbance at 380 nm), which have opposite trends to those of the Z-isomer, indicate that from the *E*-3-isomer in the presence of F^−^ ions an N-anion (*Z-3-A_1_h*) may be formed, directly cleaving NH hydrogen from the hydrazone fragment. The observed decrease in absorbance (Figure 8b) versus time corresponds to the transformation of N-anion (*E-3-A_1_h*) to Z-3-isomer. At TBAF concentration c_TBAF_ > 1 × 10^−4^ mol dm^−3^ arises *Z-y*-isomer anion (*Z-y-A_1_h*) via anion (*Z-y-A_1_i*) (y = 1,3,4) (Scheme 7). On UV-Vis spectra, this results in the formation of a long-wavelength band. 

**Figure 9:**
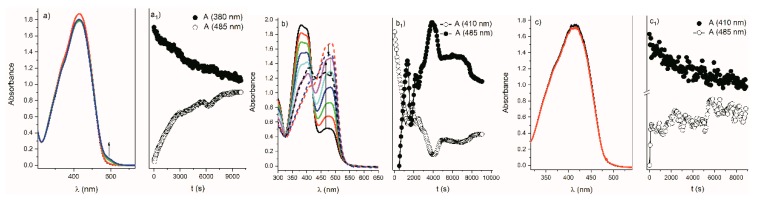
Hydrazone **3** reaction kinetics in the TBAF presence in DMSO: (**a**), (**a_1_**) initial concentration TBAF c_TBAF_ = 1 × 10^−3^ mol dm^−3^; (**b**), (**b_1_**) TBAF concentration increased to c_TBAF_ = 2 × 10^−2^ mol dm^−3^; (**c**), (**c_1_**) TBAF diluted to c_TBAF_ = 1 × 10^−3^ mol dm^−3^.

Its intensity is dependent on the TBAF concentration (Figure 6, Figure 7, and Figure 8). If thermal (*Z-y-A_1_h*)–(*E-y-A_1_h*) (y = 1,3,4) isomerization does not occur, the intensity of this absorption band would not change with time. During heat-stimulated anion (*Z-y-A_1_h*)–(*E-y-A_1_h*) (y = 1,3,4) isomerization the anion (*Z-y-A_1_h*) concentration decreases with the length of the reaction time. At such a change in concentration (*Z-y-A_1_h*) chemical equilibrium ceases (*Z-y*) = (*Z-y-A_1_i*) = (*Z-y-A_1_h*) (Scheme 7). Recovery of the steady-state leads to a gradual decrease in the starting Z-y-isomer concentration (y = 1,3,4). By a given TBAF concentration, the sum of (*Z-y-A_1_h*) and (*E-y-A_1_h*) anion concentrations in the solution reaches a maximum value, which is reflected by the long-wavelength band intensity in the UV-Vis spectra. The described experiments show that the studied hydrazones in the presence of TBAF represent a complex process consisting of acid–base and isomerization reactions. The rate constants of these reactions are dependent on the hydrazone structure, its anion’s geometric configuration, the TBAF concentration, and the solvent dielectric constant, and its proton-donor properties. Some of these parameters are a function of reaction time. The kinetics of the processes describing the hydrazone–TBAF system (Scheme 7) confirm that this is a complex process (Figure 9 and Supplementary Figure S9). Figure 8 shows the hydrazone **3**–TBAF system kinetics. The kinetic experiment was performed at the TBAF concentration equal to 10^−3^ mol dm^−3^, and 10^−2^ mol dm^−3^. For measuring the kinetics at a TBAF concentration of 10^−2^ mol dm^−3^, we used an equilibrium reaction mixture performed at TBAF concentration equal to 10^−3^ mol dm^−3^. From these figures, it is evident that the kinetics have characteristic oscillation reaction features whose amplitude and frequency are not constant but vary with time. Immediately after the increase in TBAF concentration in the reaction mixture (Figure 9b,b_1_), (*Z-y-A_1_h*)—(*E-y-A_1_h*) isomerization occurs (Scheme 7), resulting in a sharp increase in absorbance at 485 nm. After 1300 s, the absorbance decreases rapidly over time. 

Immediately after the increase of the TBAF concentration in the reaction mixture (Figure 9b,b1), the anion concentrations of (*Z-y-A_1_i*), (*Z-y-A_1_h*), and (*E-y-A_1_h*) increased. At the same time, as the concentration of these anions increased, the concentration of unreacted TBAF decreased. When the TBAF concentration dropped to a level that no longer transforms *Z-y* to (*Z-y-A_1_h*) (Scheme 6), the absorbance at 485 nm reached a maximum (t = 1300 s, Figure 9b1). Simultaneously, at this concentration of TBAF, the anion (*E-y-A_1_h*) was transformed according to Scheme 6, and the concentration of unreacted TBAF increased. This transformation step was accompanied by a decrease in absorbance at 485 nm until the TBAF concentration was high enough to restore the formation of the (*Z-y-A_1_h*) anion and subsequently the (*E-y-A_1_h*) anion (approx. 1700 s, Figure 9b1). The absorbance at 485 nm rose again. This process was repeated as shown on the kinetic record by absorbance oscillation at 485 nm. Thus, the different reactivity of the *E*- and Z-isomers of the studied hydrazones with TBAF, the dependence of these isomers on TBAF concentration, the effect of TBAF on the kinetics of transformation and isomerization of these isomers, contributes to the oscillation course of the reaction.

TBAF is involved not only in the anions present in the reaction medium formation but also in their stabilization by various stoichiometric ratios (Supplementary Figure S10). 

**Figure 10:**
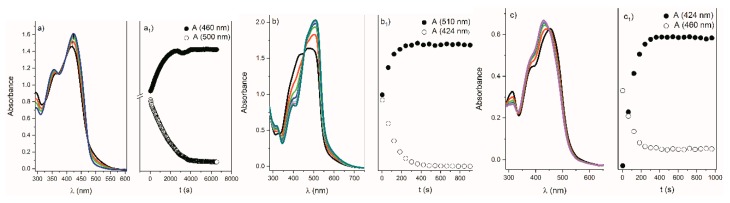
Hydrazone **4** reaction kinetics in the TBAF presence in DMSO (23 °C, 0.1 cm cuvette): (**a**), (**a_1_**) initial concentration TBAF c_TBAF_ = 1 × 10^−3^ mol dm^−3^; (**b**), (**b_1_**) TBAF concentration increased to c_TBAF_ = 5 × 10^−2^ mol dm^−3^; (**c**), (**c_1_**) TBAF diluted to c_TBAF_ = 1 × 10^−3^ mol dm^−3^.

Very weak oscillation characters in kinetics was also observed for hydrazones **1** (Supplementary Figure S9), **2** (Supplementary Figure S11), and **4** (Figure 10). The hydrazone structure affects the relative reaction rates of the entire reaction process. The individual reaction rates affect the observed oscillations amplitude and frequency.

### 2.5. Titration with TBAF—NMR Spectra

**Figure 11:**
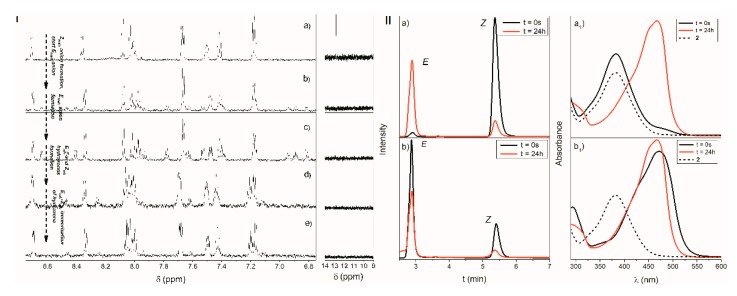
Hydrazone **2** *Z**-***isomer (1 × 10^−3^ mol dm^−3^) ^1^H-NMR spectra change in DMSO in the TBAF presence (2.5 × 10^−3^ mol dm^−3^): (**I**) (**a**) hydrazone **2** *Z*-isomer; (**b**) immediately after TBAF addition; (**c**) after 3 h since TBAF addition; (**d**) immediately after D_2_O addition; (**e**) after 24 h since D_2_O addition; (**II**) Processes visualization ongoing in the hydrazone **2** + TBAF system by UV-Vis spectra (**a_1_**), (**b_1_**), and HPLC chromatograms (**a**), (**b**).

The (*Z_m_-2-A_1_h*) and (*E_m_-2-A_1_h*) anion formation and extinction were also observed by ^1^H-NMR (Figure 11 and Supplementary Figure S12). These methods confirmed the described course of changes in the hydrazone **2** structure at the fluoride ion concentrations (2.5 × 10^−3^ mol dm^−3^) depending on time or D_2_O addition (Scheme 5). 

By D_2_O addition (Figure 11(Id)) we have ensured the anion (*Z_m_-2-A_1_h*) and (*E_m_-2-A_1_h*) disappearance to the corresponding *Z*-2- and *E*-2-isomers of the starting hydrazone **2** in DMSO. From these spectra, both hydrazone isomers in the reaction mixture can be seen. NH hydrogen signals are not present in the spectrum due to H–D exchange. From NMR spectra (Figure 11(Ie)) it is evident that hydrazone **2** *E*–*Z* isomerization occurs after 24 h at room temperature while in the dark in DMSO. The rate constants for this reaction are in Table 2. At high TBAF concentration (2 × 10^−2^ mol dm^−3^), immediately after its addition to the hydrazone **2** solution, the (*E_m_-2-A_1_h*) anion is formed (Supplementary Figure S12b). At this fluoride ion concentration, the anion (*Z_m_-2-A_1_h*) formation processes and the anions (*Z_m_-2-A_1_h*)–(*E_m_-2-A_1_h*) isomerization processes are so fast, that only the spectrum of anion (*E_m_-2-A_1_h*)**** can be registered after the time required for sample preparation and NMR spectrum measurement (Supplementary Figure S12b), which does not change with time (Supplementary Figure S12c). UV-Vis spectra and HPLC chromatograms (Figure 11II) also confirm the isomerization rate of hydrazone anions dependence on TBAF concentration. 

**Figure 12:**
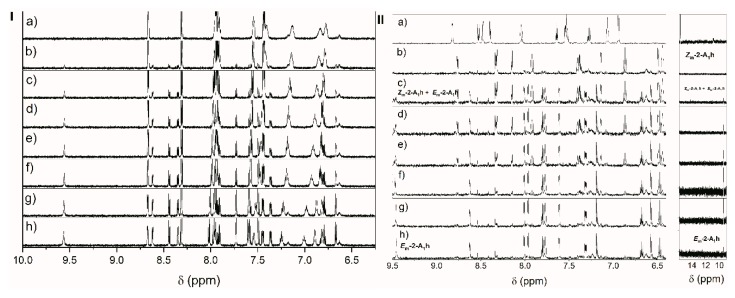
Hydrazone **1** (1 × 10^−3^ mol dm^−3^) + TBAF (1 × 10^−2^ mol dm^−3^)^1^H-NMR spectra thermal change depending on the time in DMSO: (**I**) (**a**) 10 min; (**b**) 20 min; (**c**) 40 min; (**d**) 60 min; (**e**) 80 min; (**f**) 100 min; (**g**) 200 min; (**h**) 300 min; (**II**) ^1^H-NMR spectra of hydrazone **3** (1 × 10^−3^ mol dm^−3^) + TBAF (1 × 10^−2^ mol dm^−3^) thermal change depending on time in DMSO; (**a**) 0 min; (**b**) 25 min; (**c**) 35 min; (**d**) 45 min; (**e**) 100 min; (**f**) 300 min; (**g**) 600 min; (**h**) 24 h.

The anion (*Z-y-A_1_h*) (y = 1,3) to the anion (*E-y-A_1_h*) (y = 1,3) thermal isomerization process was the same for compounds **1** and **3** (Figure 6 and Figure 7) in the presence of TBAF, as was isomerization of anion (*Z_m_-2-A_1_h*) to the anion (*Z_m_-2-A_1_h*) isomerization process for hydrazone **2** (Figure 6b). For hydrazone **1** and **3**, in the first reaction step, immediately after the addition of TBAF, N-anion (*Z-y-A_1_i*) (y = 1,3) was formed, which was transformed into the anion (*Z-y-A_1_h*) (y = 1,3) (Scheme 6 and Scheme 7). In Figure 12, there are shown processes of the (*Z-y-A_1_i*) (y = 1,3) and (*Z-y-A_1_h*) anion formation, as well as the (*Z-y-A_1_h*) (y = 1,3) to (*E-y-A_1_h*) (y = 1,3) isomerization, by the ^1^H-NMR for hydrazone **1** or **3**, in the presence of TBAF at different reaction times. For both hydrazones with increasing reaction time, the signal formation with the chemical shift close to 9.5 ppm was observed, which corresponded to (*E-y-A_1_h*) (y = 1,3).

**Figure 13:**
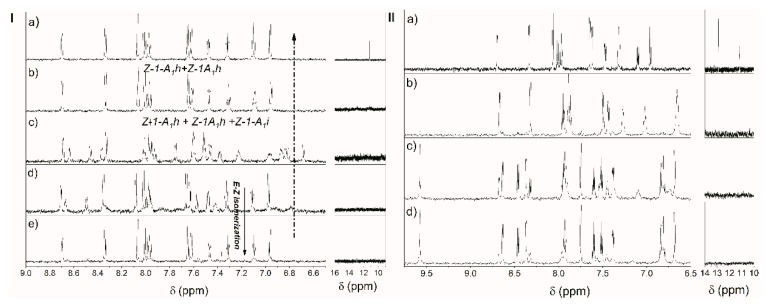
^1^H-NMR spectra of hydrazone **1** thermally stimulated change (1 × 10^−3^ mol dm^−3^) + TBAF in DMSO: (**I**) (**a**) without TBAF; (**b**) with TBAF (5 × 10^−5^ mol dm^−3^); (**c**) solution b) after 3 h; (**d**) solution c) + CD_3_OD; e) solution d) after 24 h; (**II**) Thermally stimulated ^1^H-NMR spectra change of hydrazone **1** (1 × 10^−3^ mol dm^−3^) + TBAF in DMSO; (**a**) without TBAF; (**b**) with TBAF (5 × 10^−2^ mol dm^−3^); (**c**) solution (**b**) after 1 h; (**d**) solution (**c**) after 24 h. NH groups corresponding signals are weak.

For hydrazone **3** in the initial reaction phase, this signal was a poorly resolved doublet. Doublet signal was no longer observable in the last spectrum record (Figure 12(IIh)). We assume that one signal corresponds to the anion (*E-yA_1_h*) (y = 1,3) and the other to the anion (*Z-y-A_1_h*) (y = 1,3) (Figure 12II). For hydrazone **1** the (*Z-1-A_1_h*)–(*E-1-A_1_h*) anion isomerization rate was almost 3.5 times higher than the reverse (*Z-1-A_1_h*)–(*E-1-A_1_h*) isomerization (Table 2.). This leads to the faster (*Z-1-A_1_h*) anions conversion and conversely, to an increase of (*E-1-A_1_h*) concentration with increasing reaction time. For hydrazone **3**, both anion formation and extinction rates were higher than those for hydrazone **1**. Therefore, their presence on the NMR spectrum in Figure 12(IIc–f) was observed. The TBAF concentration effect on the anion formation and their transformation is shown in Figure 13. At the TBAF concentration (5 × 10^−5^ mol dm^−3^) lower than the hydrazone **1** concentration (1 × 10^−3^ mol dm^−3^), the (*E-1-A_1_i*) concentration in the solution was so low that it could not be registered on the NMR spectra (Figure 13(Ib)).

We assume that the intensity of the decreasing signals was caused by the rapid hydrogen exchange. The low (*E-1-A_1_i*) concentration and high (*Z-1-A_1_h*)–(*E-1-A_1_h*) isomerization rate allowed suitable (*E-1-A_1_h*) anion identification alongside the hydrazone **1** *Z*-isomer (Figure 13(Ic)). 

**Figure 14:**
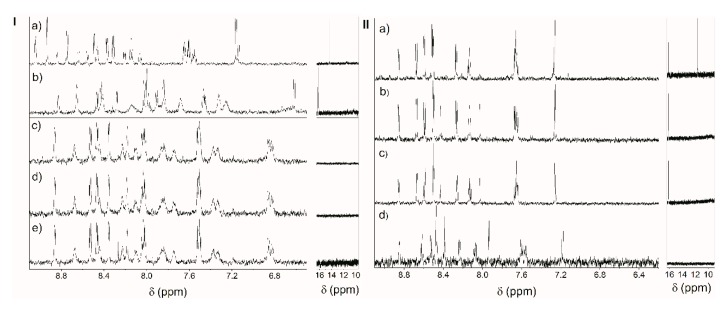
TBAF effect on hydrazone **4** ^1^H-NMR spectrum in DMSO; (**I**) (**a**) hydrazone **4** mixture of *E-* and *Z-*isomers (1 × 10^−3^ mol dm^−3^); (**b**) with TBAF 5 × 10^−3^ mol dm^−3^; (**c**) solution (**b**) after D_2_O addition; (**d**) solution (**c**) after 3 h; solution (**d**) after 24 h; (**II**) (**a**) *Z*-isomer (1 × 10^−3^ mol dm^−3^); (**b**) after TBAF 2.5 × 10^−3^ mol dm^−3^; (**c**) solution (**b**) after 3 h; (**d**) solution (**c**) after D_2_O addition.

Experimental results show that the *E*- and *Z-*isomers reactivity with TBAF were different. At the TBAF concentration approximately equal to the hydrazone *Z*-isomer concentration, the isatin NH hydrogen signal disappearance was observed in the ^1^H-NMR spectrum (Figure 14II). This signal could not be detected because the bond between the nitrogen and hydrogen disappeared either due to the anion F^−^ or due to the strong NH interaction with the basic anion. ^1^H-NMR spectrum did not change with time. If TBAF to the *E-* and *Z*-isomers mixturewas added, in the ^1^H-NMR spectrum, the other signals corresponding to the hydrazone *E*-isomer alongside with the NH signals also disappeared (Figure 14(Ia,Ib)). The characteristic signal could be observed in the spectrum at 6.6 ppm for the *Z*-isomer anion (Figure 14(Ib)) and analogous to the high TBAF concentration (Supplementary Figure S13). The *Z-*isomer did not have this signal in the NMR spectrum at low TBAF concentration.

### 2.6. Photochemical Reactivity of Studied Hydrazone N-anions 

**Figure 15:**
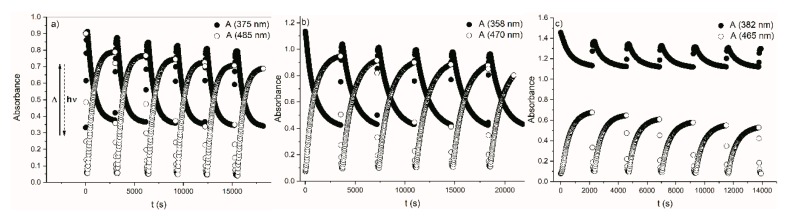
Photochemical (465 nm) and thermal (50 °C) stimulation of hydrazones E–Z or Z–E, isomerization in DMF: (**a**) hydrazone **3** (5 × 10^−5^ mol dm^−3^) in the presence of TBAF (5 × 10^−3^ mol dm^−3^); (**b**) hydrazone **1** (5 × 10^−5^ mol dm^−3^) in the presence of TBAF (1 × 10^−3^ mol dm^−3^); (**c**) hydrazone **2** (5 × 10^−5^ mol dm^−3^) in the presence of TBAF (5 × 10^−4^ mol dm^−3^).

The (*Z-y-A_1_h*), (*E-y-A_1_h*), (y = 1,3,4), or (*Z_m_-2-A_1_h*) and (*E_m_-2-A_1_h*) anions characteristic photochemical reactions are the photochemical E–Z isomerization. The thermal and photochemical Z–E anion isomerization rates are similar, and therefore, it is difficult to distinguish the photochemical reaction from a thermal reaction. Photochemical anion *E*–*Z* isomerization and thermal Z–E isomerization can be easily monitored by UV-Vis spectroscopy (Figure 15).

**Figure 16:**
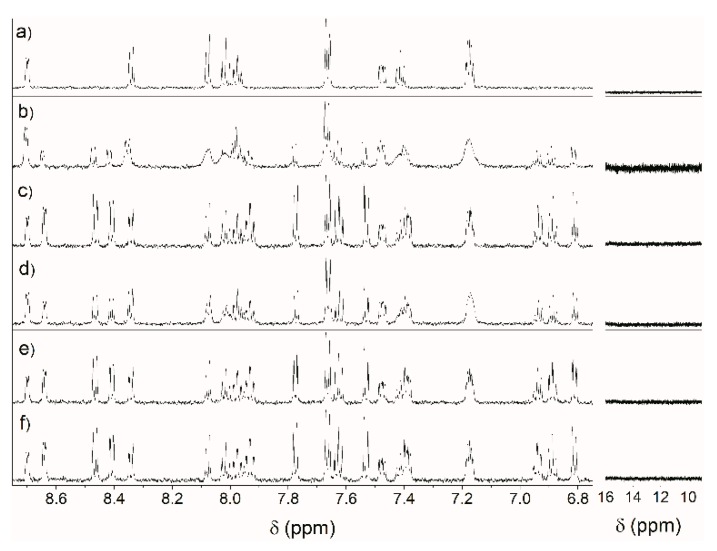
Thermally and photochemically stimulated change of hydrazone **2** ^1^H-NMR spectra (1 × 10^−3^ mol dm^−3^) + TBAF in DMF: (**a**) without TBAF; (**b**) with TBAF (2.5 × 10^−3^ mol dm^−3^); (**c**) solution (**b**) after 3 h; (**d**) solution (**c**) after irradiation 20 min, λ = 465 nm; (**e**) solution (**c**) after 3 h; (**f**) solution (**c**) after 24 h.

By thermal and photochemical stimulation alternating *Z*–*E* or *E*–*Z* isomerization repetition, ON/OFF anion functionality could be observed (Figure 15). However, for all studied anions, the switching system stability was not high enough for practical use. After each ON/OFF cycle, the switching range was reduced (Figure 15b). The ON/OFF functionality was also observed by NMR spectroscopy at room temperature (Figure 16). From Figure 16c,d, the change in signal intensity corresponding to the (*E_m_-2-A_1_h*) anion during photochemical and thermal stimulation is evident.

**Figure 17:**
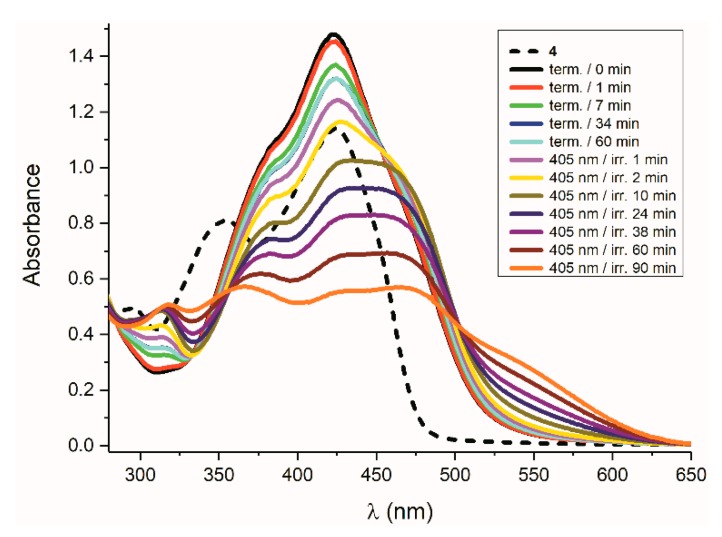
Hydrazone **4** (1 × 10^−4^ mol dm^−3^) UV-Vis spectra change in the presence of TBAF (1 × 10^−3^ mol dm^−3^) during thermic (24 °C) and photochemical reaction in DMF.

The comparison of spectra in Figure 16e,f do not indicate, that after 24 h in the dark, (*E_m_-2-A_1_h*) anion degradation occurred. Hydrazone **4** anions are the least photostable (Figure 17). After irradiation for 2.5 h, the reaction mixture no longer contained hydrazone **4** or its anions. The photoreaction product structures were not analyzed.

## 3. Materials and Methods

### 3.1. General Information

All chemicals used for synthesis were purchased from Sigma–Aldrich (St. Louis, MO, USA) or Fluorochem Ltd. (Derbyshire, UK). Solvents were dried and purified by standard methods prior to use. 6-hydrazino-2,2’-bipyridine was prepared according described procedure [21]. The samples were irradiated in the device’s own construction directly in the spectrometer’s cell by means of LEDs (electrical input 30 to 120 mW). Flash chromatography was performed on Merck (Darmstadt, Germany) silica gel 60. 

### 3.2. Synthesis

#### 3.2.1. Synthesis of Hydrazones **1** and **2**

A mixture of isatin or 1-methylisatin (1.5 mmol) and 6-hydrazino-2,2’-bipyridine (279 mg, 1.5 mmol) in ethanol (30 mL) was heated to reflux for 6 h. After cooling, the resulting solid was filtered, washed with cold ethanol (10 mL), and dried. 

*(Z)-3-(2-([2,2’-bipyridin]-6-yl)hydrazono)indolin-2-one* (**1**) yellow solid (238 mg, 74%): ^1^H-NMR (600 MHz, DMSO): δ 12.89 (s, 1H), 11.14 (s, 1H), 8.68 (dd, *J* = 4.7, 0.8 Hz, 1H), 8.31 (d, *J* = 7.9 Hz, 1H), 8.05 (d, *J* = 7.5 Hz, 1H), 7.99 (t, *J* = 7.8 Hz, 1H), 7.95 (td, *J* = 7.7, 1.8 Hz, 1H), 7.61 (dd, *J* = 16.2, 7.7 Hz, 2H), 7.45 (ddd, *J* = 7.4, 4.8, 1.0 Hz, 1H), 7.30 (td, *J* = 7.7, 1.0 Hz, 1H), 7.08 (t, *J* = 7.5 Hz, 1H), 6.94 (d, *J* = 7.8 Hz, 1H). ^13^C-NMR (151 MHz, DMSO): δ 163.66, 154.99, 154.48, 141.16, 140.36, 137.81, 130.45, 130.04, 124.77, 122.59, 120.99, 119.79, 115.53, 111.22, 108.06. Elem. anal. calcd. for C_18_H_13_N_5_O: C 68.56, H 4.16; N 22.21. Found: C 68.40; H 4.0; N 21.72. IR (ATR) ν/cm^−1^: 3065, 1699, 1681, 1433, 775, 738. m.p.: 307 °C.

*(Z)-3-(2-([2,2’-bipyridin]-6-yl)hydrazono)-1-methylindolin-2-one* (**2**) light orange solid (375 mg, 76%): ^1^H-NMR (600 MHz, CDCl_3_): δ 12.85 (s, 1H), 8.67 (ddd, *J* = 4.7, 1.8, 0.9 Hz, 1H), 8.44 (dt, *J* = 8.0, 1.0 Hz, 1H), 8.09 (dd, *J* = 7.6, 0.7 Hz, 1H), 7.88–7.78 (m, 2H), 7.68 (dd, *J* = 7.4, 0.5 Hz, 1H), 7.65 (d, *J* = 8.1 Hz, 1H), 7.36–7.28 (m, 2H), 7.14 (td, *J* = 7.5, 0.8 Hz, 1H), 6.90 (d, *J* = 7.8 Hz, 1H), 3.33 (s, 3H). ^13^C-NMR (151 MHz, CDCl_3_): δ 161.80, 155.65, 154.95, 154.55, 149.01, 141.80, 139.01, 136.85, 128.87, 128.63, 123.68, 122.69, 121.19, 120.89, 119.35, 115.47, 108.50, 108.03, 25.56. Elem. anal. calcd. for C_19_H_15_N_5_O: C 69.29, H 4.59; N 21.26. Found: C 69.11; H 4.41; N 20.65. IR (ATR) ν/cm^−1^: 3053, 1681, 1548, 1426, 773, 743. m.p.: 210 °C.

#### 3.2.2. Synthesis of Hydrazones **3** and **4**

Trifluoroacetic acid (0.75 mL, 0.009 mol) was added to a cooled (0 °C) solution (4.5 mL CH_2_Cl_2_) of 3-amino-2,2’-bipyridine (0.513 g, 0.003 mol), followed by isoamyl nitrite (0.61 mL, 0.004 mol). After 1 h, the diazonium salt of 3-amino-2,2’-bipyridine was precipitated out by cooling the reaction mixture to –78 °C, followed by the addition of diethyl ether (30 mL). The precipitate was filtered. A sample (2 mmol; 0.27 g) of indol-2-one was dissolved in dilute potassium hydroxide solution (15 mL) and cooled in a salt⁄ice bath and then cold diazonium solution was added to this cooled solution portion wise by stirring. The solution was further stirred at 0–5 °C for 1 h. The pH of the reaction mixture was maintained at 4–6 by the addition of solid sodium acetate in portions. The mixture was stirred for 24 h at room temperature. The resulting solid was filtered, washed with cold water, and air-dried. Crystallization from ethanol gave yellow crystalline.

*(Z)-3-(2-(2,2’-bipyridin-3-yl)hydrazono)indolin-2-one* (**3**), yellow solid (328 mg, 52%): ^1^H-NMR (600 MHz, DMSO): δ 15.70 (s, 1H), 10.89 (s, 1H), 8.80 (d, *J* = 4.0 Hz, 1H), 8.50 (d, *J* = 8.1 Hz, 1H), 8.45 (dd, *J* = 8.4, 1.3 Hz, 1H), 8.37 (dd, *J* = 4.3, 1.4 Hz, 1H), 8.04–7.98 (m, 1H), 7.62 (d, *J* = 7.4 Hz, 1H), 7.54–7.46 (m, 1H), 7.25 (dd, *J* = 7.6, 6.7 Hz, 1H), 7.04 (t, *J* = 7.2 Hz, 1H), 6.92 (d, *J* = 7.7 Hz, 1H). ^13^C-NMR (151 MHz, DMSO) δ 161.83, 156.74, 147.04, 142.28, 140.39, 139.61, 137.59, 137.13, 130.36, 128.95, 125.16, 123.13, 121.90, 121.74, 121.61, 121.27, 119.08, 110.29. Elem. anal. calcd. for C_18_H_13_N_5_O, C 68.56, H 4.16, N 22.21. Found: C 68.48, H 4.09, N 21.88. IR (ATR) ν/cm^−1^: 3180, 3047, 1693, 1464, 1164, 798. m.p: 238–240 °C.

*(Z)-3-(2-(2,2’-bipyridin-3-yl)hydrazono)-5-nitroindolin-2-one* (**4**), (339 mg, 47%): ^1^H-NMR (600 MHz, DMSO): δ 15.97 (s, 1H), 8.84 (s, 1H), 8.64 (d, *J* = 8.0 Hz, 1H), 8.56 (d, *J* = 8.3 Hz, 1H), 8.46 (s, 1H), 8.20 (d, *J* = 11.0 Hz, 1H), 8.07 (d, *J* = 7.3 Hz, 1H), 7.59–7.51 (m, 2H), 7.13 (d, *J* = 8.0 Hz, 1H). ^13^C-NMR (151 MHz, DMSO) δ 162.67, 157.10, 147.59, 143.84, 142.78, 139.78, 138.35, 138.05, 128.83, 125.85, 125.29, 123.93, 123.29, 122.59, 122.38, 114.66, 110.93, 110.92. Elem. anal. calcd. for C_18_H_12_N_6_O_3_: C 60.00, H 3.36, N 23.32. Found: C 59.36, H 3.11, N 22.88. IR (ATR) ν/cm^−1^: 3066, 1731, 1449, 1338, m.p.: > 350 °C.

### 3.3. Spectroscopic Measurements

NMR spectra were recorded in 5 mm NMR tubes on a VNMRS 600 MHz spectrometer (600 MHz for ^1^H; 150 MHz for ^13^C, Varian, Inc., Palo Alto, CA, USA) in DMSO-*d*_6_ and CDCl_3_ as solvents (c_sensor_ = 1 × 10^−4^; anion concentration up to 30 equivalents). Chemical shifts were referenced to tetramethylsilane (TMS) as an internal standard. Attenuated total reflection Fourier transform infrared (ATR-FTIR) spectra of all the described experiments were measured on a Nicolet 6700 FTIR (from ThermoNicolet Corp., Madison, WI, USA). Spectra were recorded with ATR mathematical corrections yielding a 1.0 cm^−1^ actual resolution and 40 measurements were averaged. Spectra for liquid samples were measured in a cell with CaF_2_ windows and a path length of 0.2 mm. Electronic absorption spectra were obtained on a HP 8452A diode array spectrophotometer (Hewlett Packard, Palo Alto, CA, USA). Solution fluorescence was measured in a 1 cm cuvette with an FSP 920 (Edinburgh Instruments, Edinburgh, UK) spectrofluorimeter in a right-angle or front-face arrangement (to exclude solution self-absorption). The solvents used (CHCl_3_, DMSO, DMF) were HPLC (CHCl_3_; LiChrosolv^®^, Merck, Darmstadt, Germany) or UV-spectroscopy grade (DMSO and DMF; Uvasol^®^, Merck) and were used without further purification. Detailed determination of the association constant for apparent 1:1 complex is described in ESI Association constant determinations.

### 3.4. HPLC Chromatography

HPLC chromatography was carried out using a chromatographic system (Agilent Technologies, Santa Clara, CA, USA) consisting of a quaternary pump (G 1311A), variable wavelength detector VWD G 1314A), manual injector (Rheodyne model 7725i) with 20 μL sample loop, and a degasser (g1379A) all the 1100 series. For analyses of compounds **1** and **2**, column ZORBAX SB-Phenyl (150 mm × 4.6 mm i.d), mobile phase A 0.1% HCOOH (formic acid), mobile phase B 55% CH_3_CN (acetonitrile), and 0.1% HCOOH, at the flow rate of 1 mL min^−1^ and detection at 390 nm, was used. For analyses of compounds **3** and **4**, column Lichrosphere 100 RP-18e (250 mm × 4.0 mm i.d), mobile phase A water, mobile phase B CH_3_CN with the gradient 0,0 min. 55% CH_3_CN and 11 min. 99% CH_3_CN, at the flow rate of 1 mL min^−1^ and detection at 390 nm, was used.

### 3.5. Quantum Chemical Calculations

The relative stabilities of studied hydrazones were investigated using quantum-chemical calculations at the M062x 6-31+g(dp) level. Stationary points were characterized as minima by computation of harmonic vibrational frequencies. The zero-point vibrational energies and thermal corrections to the free energies were determined by using the unscaled M062 6-31+g(dp) frequencies. Free energies of solvated structures were calculated using the integral equation formalism-polarizable continuum model (IEF-PCM) method. All calculations were performed by Gaussian 16 program package [22].

## 4. Conclusions

Four new hydrazones with bipyridyl fragments were synthesized. Hydrazone *Z*-isomers exhibit high thermal stability. The *E-*isomers formation at 80 °C in DMF was not observed. The characteristic photochemical reaction for all studied hydrazones is the *Z*–*E* isomerization in CHCl_3_. Isomerization reactions are photoreversible reactions. The photoreaction reversibility is achieved by appropriately selected light wavelengths used for irradiation. The photoreaction products vary depending on the hydrazone structures in DMF. By irradiation in DMF hydrazones, **3** and **4** undergo *Z*–*E* isomerization similar to CHCl_3_. After irradiation of hydrazone **1** and **2** in DMF the photoreaction product is a tautomer (Scheme 4). When using light with the appropriate wavelength, the photo-tautomerization reaction is reversible. In these conditions the studied hydrazones have ON/OFF switch properties. The thermally stimulated tautomerization occurs at a temperature above 70 °C. Only from the hydrazones **1** and **2** *E*-isomers is a tautomer formed. The photoreaction or thermal reaction results in the corresponding hydrazone *E*-isomer, which is immediately subjected to tautomerization. Z-isomers are not subjected to this tautomerization. In the presence of fluoride ions, NH hydrogen from the studied hydrazones is cleaved, and the corresponding anions are formed. The hydrazone structure determines the relative NH hydrogen acidity in the molecule. For hydrazones **1** and **3**, the isatin NH hydrogen is preferably cleaved from the hydrazone molecule. Thus, the formed anion is transformed into the anion, which has a negative charge located at the nitrogen hydrazone part. For the hydrazone **2** under the same conditions, the anion forms by direct NH hydrogen cleavage from the hydrazone fragment. The resulting anion *Z*-isomers are isomerized at room temperature. The corresponding anion’s *E*-isomers are formed in high yields. From the measured data it is evident that the anion’s *Z*–*E* isomerization is faster than the corresponding anion’s *E*–*Z* isomerization. Anion isomerization represents a complex reaction scheme. Kinetic records of these isomerization courses have characteristic oscillation reaction features. Anions from *E*-isomers are photochemically isomerized to the corresponding hydrazone’s *Z*-isomers. By alternating heat and light stimulation, it is possible to control the anion isomerization process reversibly. In this switching, the anions present in the reaction medium are also degraded.

## Figures and Tables

**Table 1:** Maxima UV-Vis, fluorescence spectra, and photophysical parameters of studied hydrazones.

Compound	UV-Vis		Fluorescence							
	DMF	CHCl_3_	DMF				CHCl_3_			
	λ_max_ (nm) log(ε) (l mol cm^−1^)	λ_max_ (nm) log(ε) (l mol cm^−1^)	λ_em_/λ_exc_ (nm)	Stokes Shift (cm^−1^)	τ (ns)	ɸ_F_	λ_em_/λ_exc_ (nm)	Stokes Shift (cm^−1^)	τ (ns)	ɸ_F_
**1**	383 4.40	391 4.22	557/383	8156	τ^1^ = 0.13 (80.4%) τ^2^ = 7.69 (19.6%) χ^2^ = 0.97	<0.01	521/390	6382	τ^1^ = 0.17 (89.9%) τ^2^ = 5.54 (10.1%) χ^2^ = 0.92	<0.01
**2**	382 4.38	386 4.38	464/383	4626	τ^1^ = 0.12 (80%) τ^2^ = 8.17 (20%) χ^2^ = 1.16	<0.01	561/390	8081	τ^1^ = 0.26 (74%) τ^2^ = 7.82 (26%) χ^2^ = 1.3	<0.01
**3**	414 4.40	422 4.37	490/412	3746	τ^1^ = 4.21 (93.6%) τ^2^ = 9.51 (6.4%) χ^2^ = 1.33	0.04	483/420	2993	τ^1^ = 3.46 (29.8%); τ^2^ = 6.77 (70.2%) χ^2^ = 1.33	0.02
4 *	411 4.08	353 3.97 418 4.13	507/412 ** 640/412	8060 ** 8706	τ^1^ = 0.11 (11.8%) τ^2^ = 4.99 (72.2%) τ^3^ = 19.5 (16.0%) χ^2^ = 1.23	<0.01	480/420	3090	τ^1^ = 0.41 (4.5%) τ^2^ = 3.41 (95.5%) χ^2^ = 1.29	<0.01

ɸ_F_—Fluorescence quantum yield; τ—Fluorescence lifetime of the corresponding component; χ^2^—Quality of fluorescence lifetime fitting; %—relative fluorescence intensity of the corresponding component; * *Z*/*E* isomer mixture; ** shoulder.

**Table 2:** *Z*/*E* isomer ratio in photostationary state in DMF (λ_irr_ = 370 nm) and CHCl_3_ (λ_irr_ = 405 nm), rate constants of thermal *E*–*Z* isomerization (k*_E_*_-*Z*_) and tautomerization (k_t_) in DMF.

Compound	DMF/25 °C	CHCl_3_/25 °C	DMF/80 °C	
	[*Z*]/[*E*]	[*Z*]/[*E*]	10^5^ × k_E-Z_ [s^−1^]	10^4^ × k_t_ [s^−1^]
**1**	17.5	2.9	-	9.2
**2**	11.1	1.2	-	12.0
**3**	1.1	0.8	7.8	-
**4**	2.0	0.8	2002.0	-

**Table 3:** The studied hydrazones pK_a_ values in H_2_O: DMSO (1:9) and rate constants k^1^*_E_*_–*Z*_ and k^1^*_Z_*_–*E*_ thermal *E*–*Z* isomerization of anions in DMF at 25 °C.

Compound	pK_a1_	pK_a2_	k^1^*_E-Z_* × 10^5^ (s^−1^)	k^1^*_Z-E_* × 10^5^ (s^−1^)
**1**	8.7	9.3	4.98	17.5
**2**	-	9.6	23.49	320
**3**	8.8	9.4	7.09	78
**4**	7.8	8.6	157.03	186

## Supplementary Materials

The following are available online, Figure S1: Hydrazone **4** kinetics of heat-stimulated E–Z isomerization (5 × 10^−5^ mol dm^−3^) at 80 °C in DMF. Figure S2: UV-Vis spectra change during irradiation: (**a**) hydrazone **3** (5 × 10^−5^ mol dm^−3^) in CHCl_3_; (**b**) hydrazone **3** (5 × 10^−5^ mol dm^−3^) in DMF; (**c**) hydrazone **4** (1 × 10^−4^ mol dm^−3^) in CHCl_3_; (**d**) hydrazone **4** (1 × 10^−4^ mol dm^−3^) in DMF. Figure S3: UV-Vis spectra and chromatograms change during hydrazone **2** (5 × 10^−5^ mol dm^−3^) photolysis: (**a**) and (**b**) photolysis in CHCl_3_; (**c**) and (**d**) photolysis in DMF. Figure S4: UV-Vis spectrum change: (**a**) hydrazone **1** (1 × 10^−4^ mol dm^−3^) a light-stimulated change in CHCl_3_; (**b**) hydrazone **1** (5 × 10^−5^ mol dm^−3^) reversible light-stimulated change in DMF. Figure S5: H–D exchange in DMSO: (**a**) hydrazone **3**; (**b**) solution a + D_2_O; (**c**) solution b after 24 h; (**d**) hydrazone **1**; (**e**) solution d + D_2_O; (**f**) hydrazone **4**; (**g**) solution f + D_2_O. Figure S6: Thermic change of hydrazone **4** (1 × 10^−4^ mol dm^−3^) in the TBAF presence: (**a**), (**c**) chromatogram - c_TBAF_ = 5 × 10^−3^ mol dm^−3^; (**b**), (**d**) UV-Vis spectra - c_TBAF_ = 4 × 10^−4^ mol dm^−3^ in DMF. Figure S7: UV-Vis spectra change of the *Z*-isomer and an *E*- and Z-isomers mixture of the hydrazone **3** (5 × 10^−5^ mol dm^−3^) depending on the TBAF concentration in DMF. Figure S8: Hydrazone **3** *E*- and *Z*-isomers (1 × 10^−3^ mol dm^−3^) ^1^H-NMR spectrum change during H–D hydrogen exchange in DMSO: (**a**) Z-isomer from hydrazone **3**; (**b**) a mixture of *Z*- and *E*-isomers from hydrazone **3**; (**c**) solution b + 3 drops of D_2_O; (**d**) solution c after 1 h. Figure S9: Hydrazone **1** reaction kinetics in the TBAF presence in DMSO: (**a**), (**a_1_**) initial concentration TBAF c_TBAF_ = 1 × 10^−3^ mol dm^−3^; (**b**), (**b_1_**) TBAF concentration increased to c_TBAF_ = 5 × 10^−2^ mol dm^−3^; (**c**), (**c_1_**) TBAF diluted to c_TBAF_ = 1 × 10^−3^ mol dm^−3^. Figure S10: Job‘s plots for: (**a**) hydrazone **1**; (**b**) hydrazone **2**; (**c**) hydrazone **3**; (**d**) hydrazone **4** in DMF. Titration with TBAF. Figure S11: Hydrazone **2** reaction kinetics in the TBAF presence in DMSO: (**a**), (**a_1_**) initial concentration TBAF c_TBAF_ = 1 × 10^−3^ mol dm^−3^; (**b**), (**b_1_**) TBAF concentration increased to c_TBAF_ = 2 × 10^−2^ mol dm^−3^; (**c**), (**c_1_**) TBAF diluted to c_TBAF_ = 1 × 10^−3^ mol dm^−3^. Figure S12: *Z*-isomer of hydrazone **2** (1 × 10^−3^ mol dm^−3^) ^1^H-NMR spectra change in DMSO at TBAF (2 × 10^−2^ mol dm^−3^) presence: (**a**) hydrazone **2** *Z*-isomer; (**b**) immediately after TBAF addition *E*-isomer of hydrazone **2** formation from ***E_mah_***; (**c**) after 3 h since TBAF addition. Figure S13: System hydrazone **4** (1 × 10^−3^ mol.dm^−3^) + TBAF (1 × 10^−3^ mol.dm^−3^) ^1^H-NMR spectra changes as the time function in DMSO. Figure S14: Hydrazone **3** thermally and photochemically stimulated change of ^1^H-NMR spectrum (1 × 10^−^^3^ mol dm^−^^3^) + TBAF in DMF: (**a**) without TBAF; (**b**) with TBAF (1 × 10^−^^3^ mol dm^−^^3^); (**c**) solution b) after 3 h; (**d**) solution c) irradiated 20 min. with light 465 nm. ^1^H-NMR spectra changes as the time function in DMSO. Table S1: Calculated Gibbs energy for *E*- and *Z*-isomers of studied hydrazones and their tautomeric forms. Table S2: Calculated Gibbs energies for *E*- and *Z*-isomer anions of studied hydrazones.
