# Supplementary material for: ON/OFF Photostimulation of Isatin Bipyridyl Hydrazones: Photochemical and Spectral Study
Source: Molecules. 2019 Jul 23;24(14):2668. doi: 10.3390/molecules24142668 (PMC6680666; doi:10.3390/molecules24142668)
Supplement: Supplementary file 1 [file molecules-24-02668-s001.pdf]

# ON/OFF Photostimulation of Isatin Bipyridyl Hydrazones. Photochemical and Spectral Study.

**Róbert Šandrik [1], Pavol Tisovský [1,*], Klaudia Csicsai [1], Jana Donovalová [1], Martin Gáplovský [2], Róbert Sokolík [1], Juraj Filo [1], Anton Gáplovský [1]**

[1]Faculty of Natural Sciences, Institute of Chemistry, Comenius University, Ilkovičova 6,

Mlynská dolina CH-2, SK-842 15 Bratislava, Slovakia;

[2]Department of Pharmaceutical Chemistry, Faculty of Pharmacy, Comenius University, Odbojárov 10, SK-832 32 Bratislava

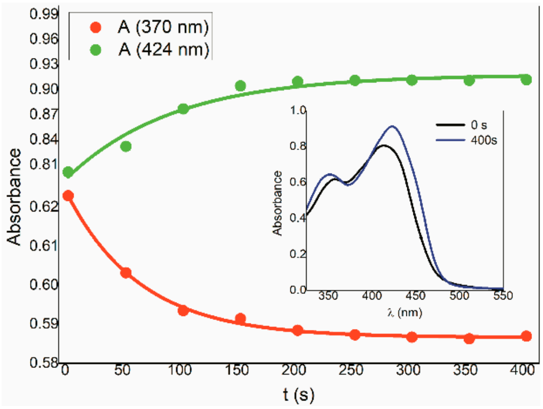


**Figure S1.** Hydrazone **4** kinetics of heat-stimulated *E-Z* isomerization (5 × 10$^{-5}$ mol dm$^{-3}$) at 80 °C in DMF. In set hydrazone **4** UV-Vis spectra during isomerization.

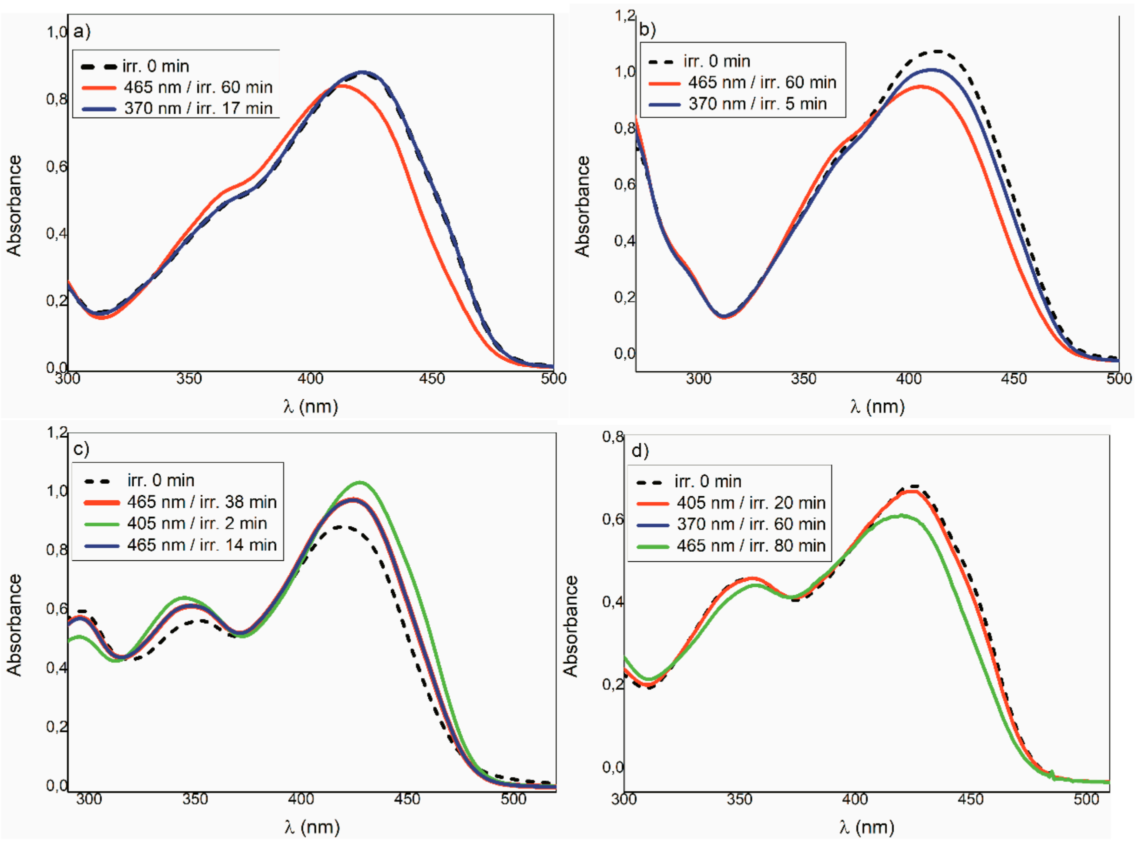

**Figure S2.** UV-Vis spectra change during irradiation: (**a**) hydrazone **3** ($5 \times 10^{-5}$ mol dm$^{-3}$) in $CHCl_3$; (**b**) hydrazone **3** ($5 \times 10^{-5}$ mol dm$^{-3}$) in DMF; (**c**) hydrazone **4** ($1 \times 10^{-4}$ mol dm$^{-3}$) in $CHCl_3$; (**d**) hydrazone **4** ($1 \times 10^{-4}$ mol dm$^{-3}$) in DMF.

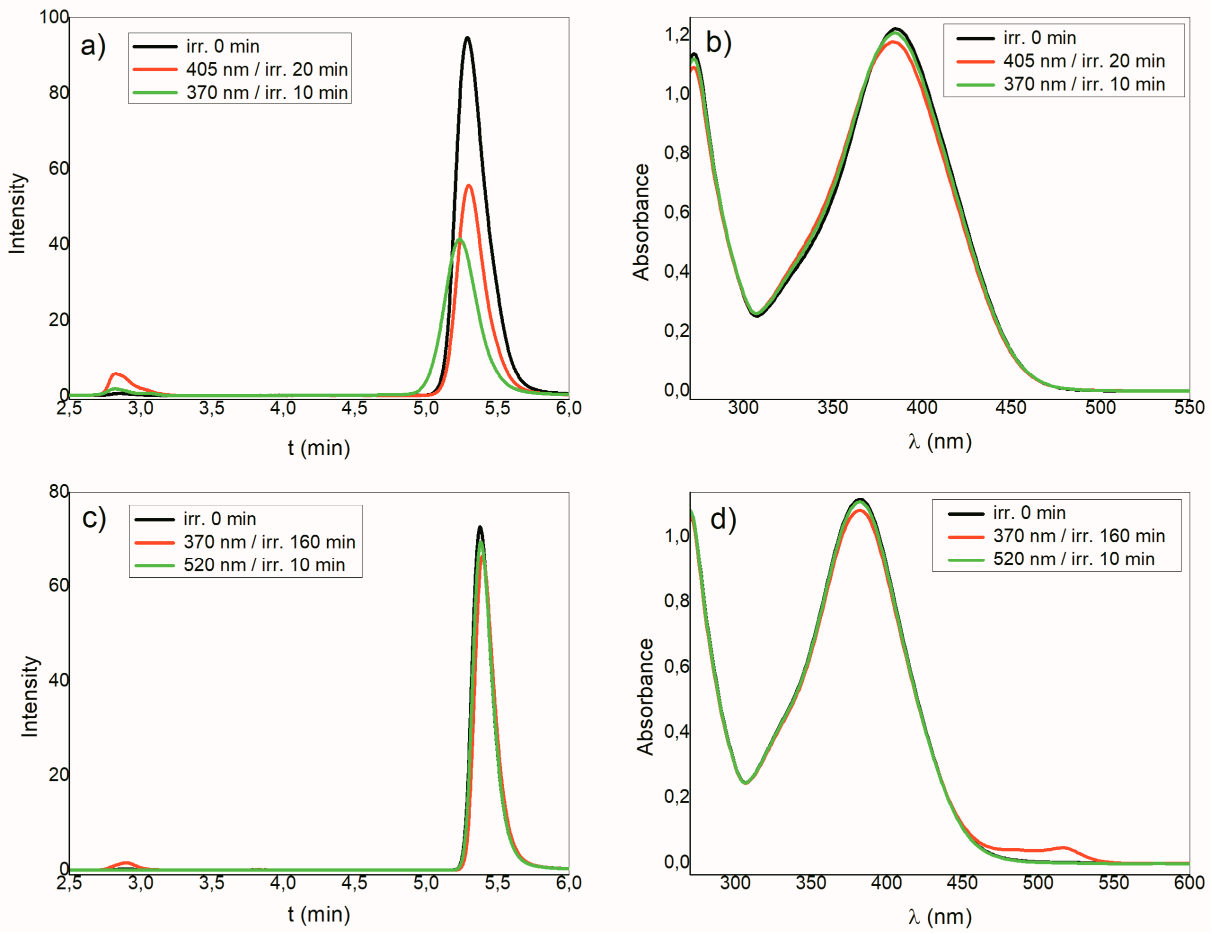


**Figure S3.** UV-Vis spectra and chromatograms change during hydrazone **2** ($5 \times 10^{-5}$ mol dm$^{-3}$) photolysis: (**a**) and (**b**) photolysis in $CHCl_3$;(**c**) and (**d**) photolysis in DMF.

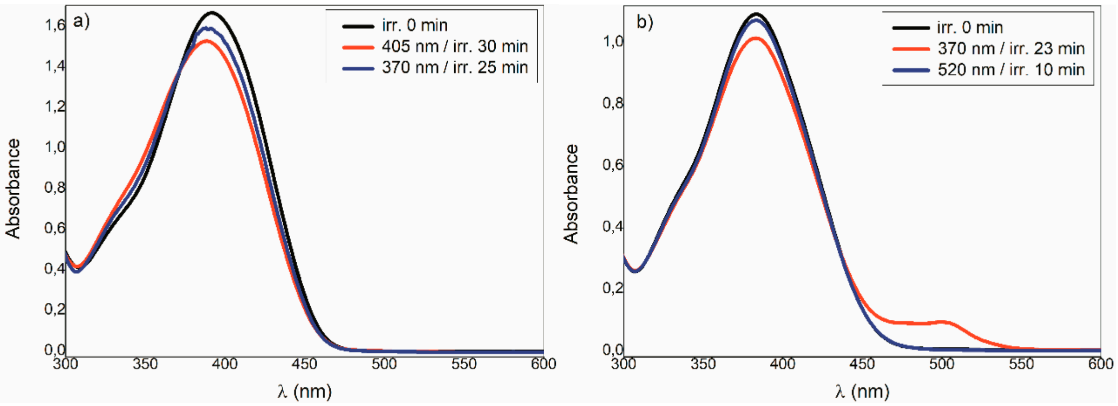


**Figure S4.** UV-Vis spectrum change: (**a**) hydrazone **1** ($1 \times 10^{-4}$ mol dm$^{-3}$) a light-stimulated change in $CHCl_3$; (**b**) hydrazone **1** ($5 \times 10^{-5}$ mol dm$^{-3}$) reversible light-stimulated change in DMF.

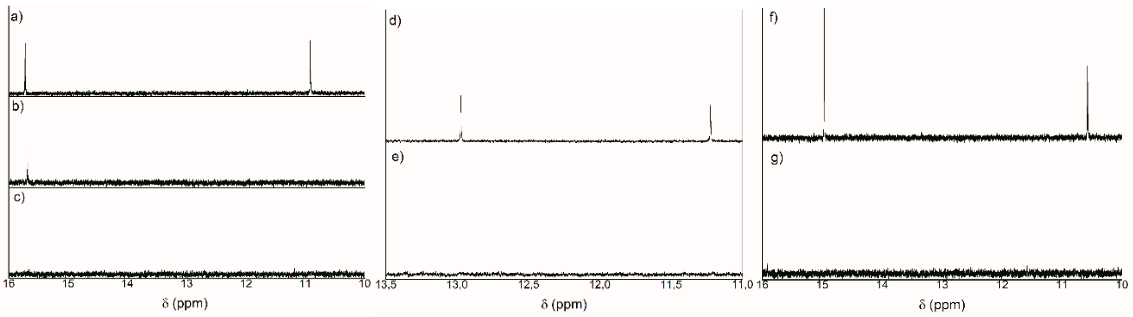


**Figure S5.** H-D exchange in DMSO: (**a**) hydrazone **3**; (**b**) solution a + $D_2O$; (**c**) solution b after 24 h; (**d**) hydrazone **1**; (**e**) solution d + $D_2O$; (**f**) hydrazone **4**; (**g**) solution f + $D_2O$.

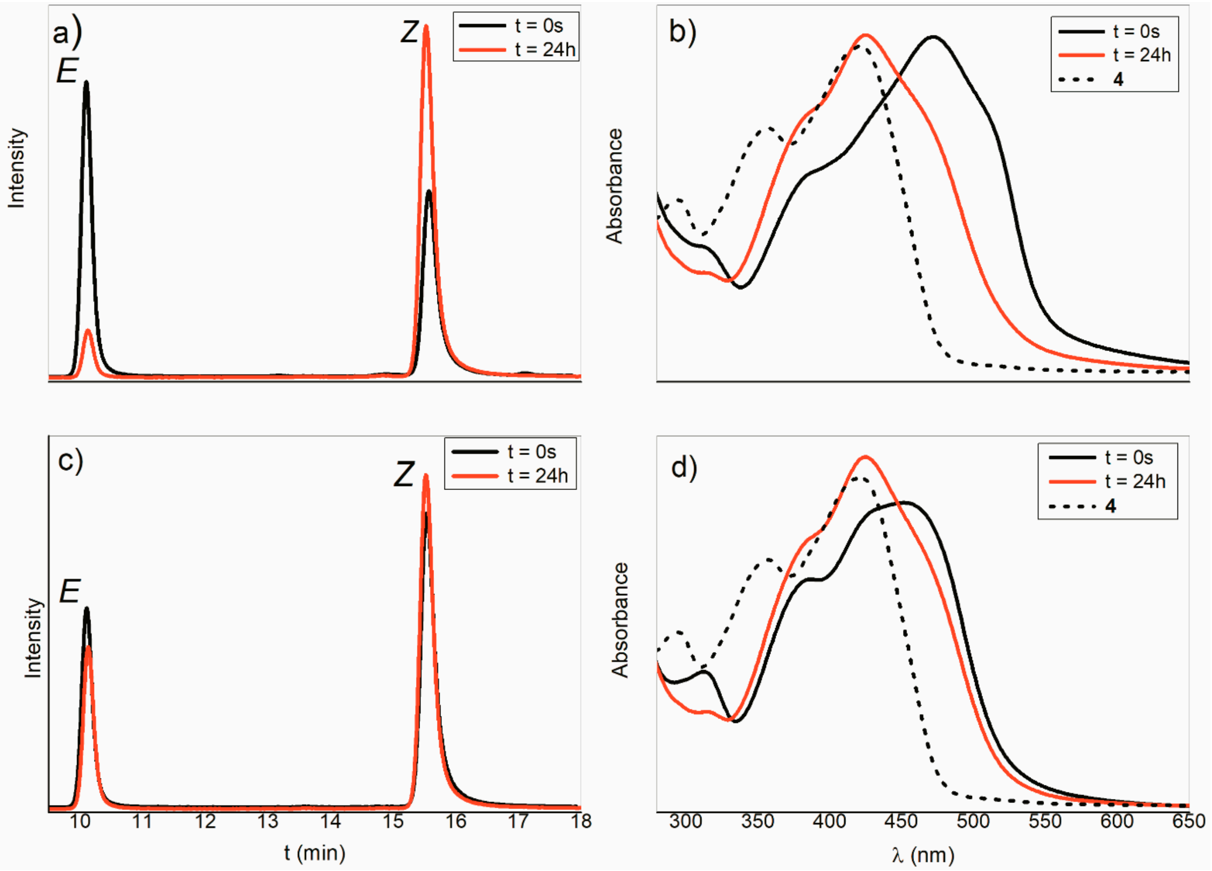


**Figure S6.** Thermic change of hydrazone **4** ($1 \times 10^{-4}$ mol dm$^{-3}$) in the TBAF presence: (**a**), (**c**) chromatogram - $c_{TBAF}$ = $5 \times 10^{-3}$ mol dm$^{-3}$; (**b**), (**d**) UV-Vis spectra - $c_{TBAF}$ = $4 \times 10^{-4}$ mol dm$^{-3}$ in DMF.

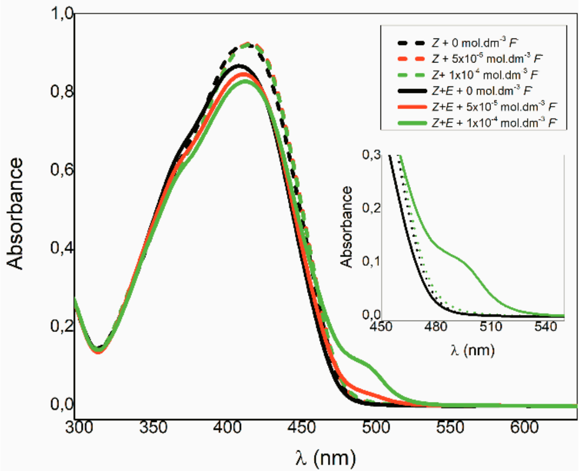


**Figure S7.** UV Vis spectra change of the *Z*-isomer and an *E* and *Z*-isomers mixture of the hydrazone **3** ($5 \times 10^{-5}$ mol dm$^{-3}$) depending on the TBAF concentration in DMF.

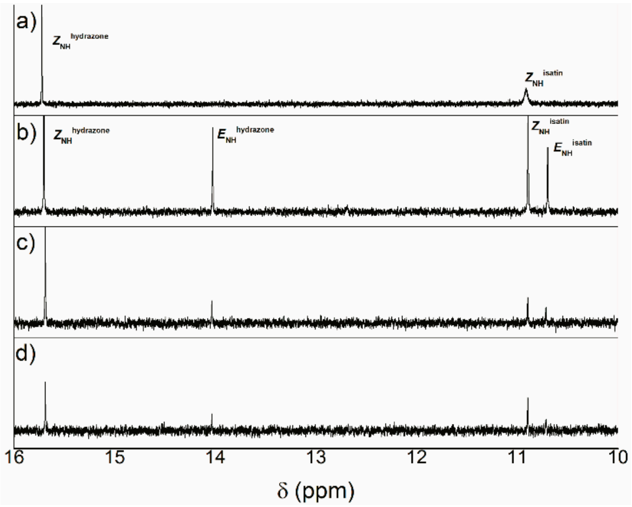


**Figure S8.** Hydrazone **3** *E* and *Z* isomers ($1 \times 10^{-3}$ mol dm$^{-3}$) $^{1}$H-NMR spectrum change during H-D hydrogen exchange in DMSO: (**a**) *Z*-isomer from hydrazone **3**; (**b**) a mixture of *Z* and *E* isomers from hydrazone **3**; (**c**) solution b + 3 drops of $D_2O$; (**d**) solution c after 1 h.

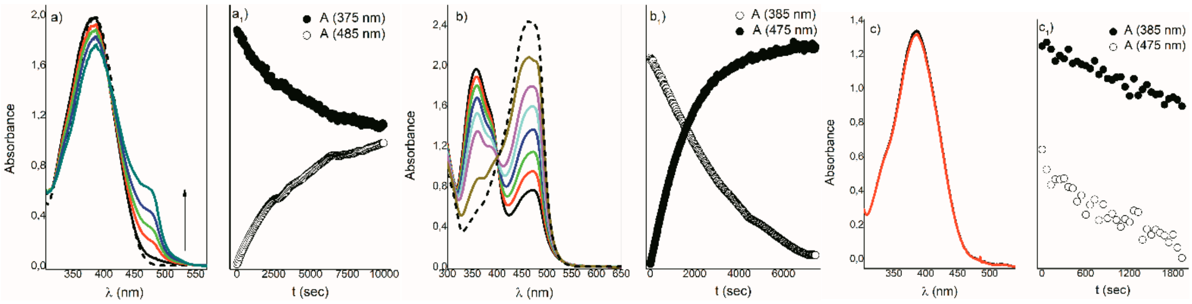


**Figure S9.** Hydrazone **1** reaction kinetics in the TBAF presence in DMSO: (**a**), (**a**$_1$) initial concentration TBAF $c_{TBAF} = 1 \times 10^{-3}$ mol dm$^{-3}$; (**b**), (**b**$_1$) TBAF concentration increased to $c_{TBAF} = 5 \times 10^{-2}$ mol dm$^{-3}$; (**c**), (**c**$_1$) -TBAF diluted to $c_{TBAF} = 1 \times 10^{-3}$ mol dm$^{-3}$.

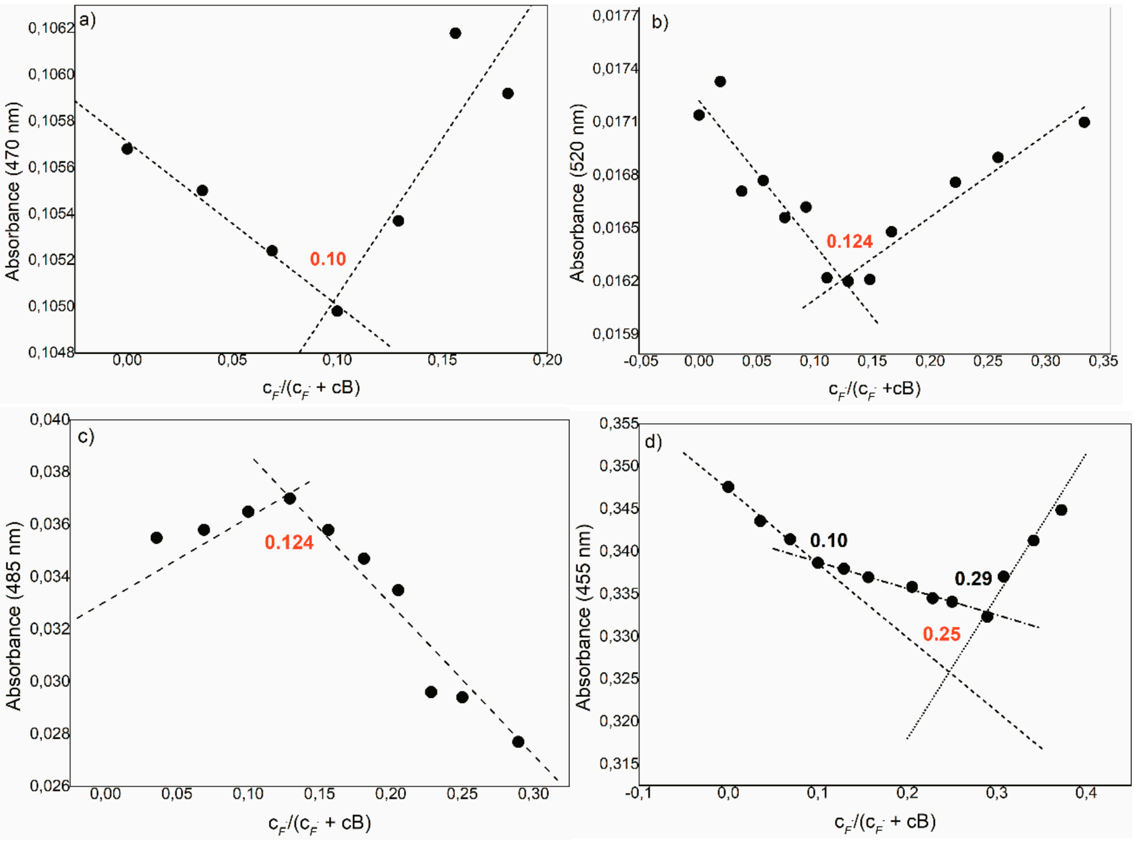


**Figure S10.** Job's plots for: (**a**) hydrazone **1**; (**b**) hydrazone **2**; (**c**) hydrazone **3**; (**d**) hydrazone **4** in DMF. Titration with TBAF.

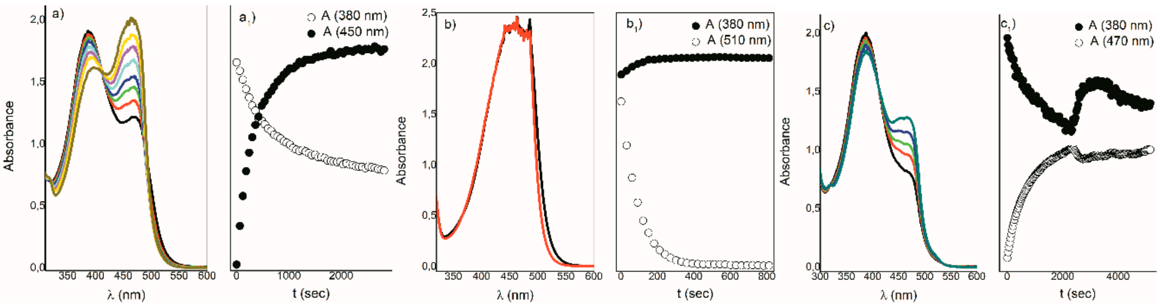


**Figure S11.** Hydrazone **2** reaction kinetics in the TBAF presence in DMSO: (**a**), (**a**1) - initial concentration TBAF $c_{TBAF} = 1 \times 10^{-3}$ mol dm$^{-3}$; (**b**), (**b**1) -TBAF concentration increased to $c_{TBAF} = 2 \times 10^{-2}$ mol dm$^{-3}$; (**c**), (**c**1) -TBAF diluted to $c_{TBAF} = 1 \times 10^{-3}$ mol dm$^{-3}$.

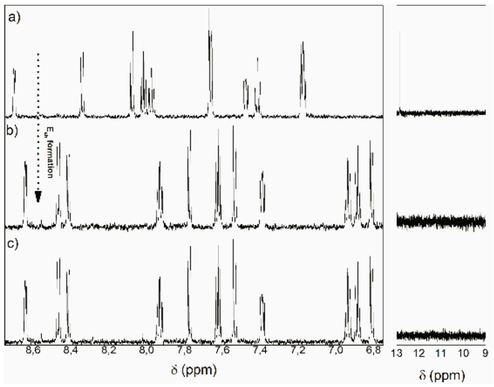

**Figure S12.** Z-isomer of hydrazone **2** (1 × 10$^{-3}$ mol dm$^{-3}$) $^{1}$H-NMR spectra change in DMSO at TBAF (2 × 10$^{-2}$ mol dm$^{-3}$) presence: (**a**) hydrazone **2** *Z*-isomer; (**b**) immediately after TBAF addition - *E*-isomer of hydrazone **2** formation from $\boldsymbol{E_{mah}}$; (**c**) after 3 h since TBAF addition.

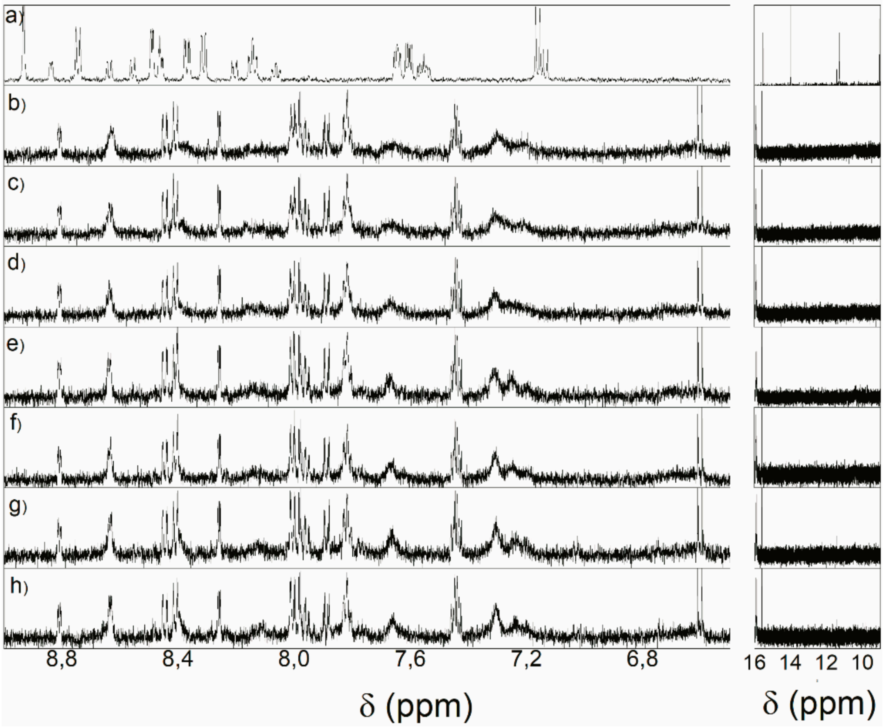


**Figure S13.** System hydrazone **4** (1 × 10$^{-3}$ mol dm$^{-3}$) + TBAF (1 × 10$^{-3}$ mol dm$^{-3}$) $^{1}$H-NMR spectra changes as the time function in DMSO.

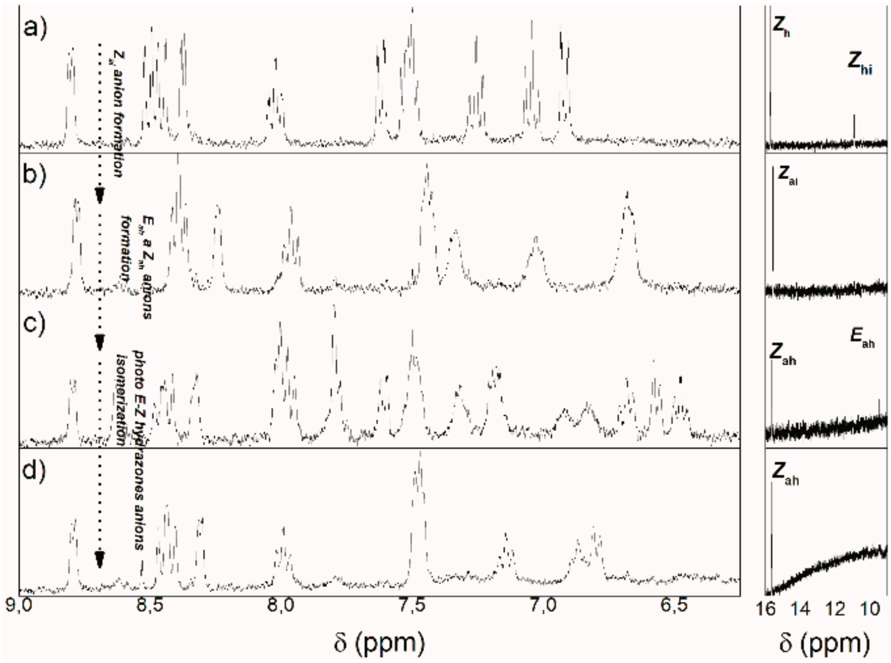


**Figure S14.** Hydrazone **3** thermally and photochemically stimulated change of $^{1}$H-NMR spectrum (1 × 10$^{-3}$ mol dm$^{-3}$) + TBAF in DMF: (**a**) without TBAF; (**b**) with TBAF (1 × 10$^{-3}$ mol dm$^{-3}$); (**c**) solution b) after 3 h; (**d**) solution c) irradiated 20 min. with light 465 nm.

**Table S1.** Calculated Gibbs energies [M062x 6-31+g(dp)] level (T = 298.15 K) for *E* and Z isomers of studied hydrazones and their tautomeric forms.

| $Z_{isomer}$ | Compound<br>1 | ΔG/ $ΔG_{DMF}$ $kJ.mol^{-1}$ | ΔG/ $ΔG_{DMF}$ $kJ.mol^{-1}$ | Compound<br>1 | $E_{isomer}$ |
|---|---|---|---|---|---|
| *Z-1* | 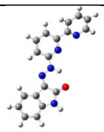 | 0/0 | 19.7/10.7 | 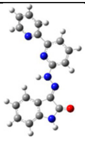 | *E-1* |
| *Z-1-c1* | 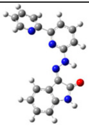 | 16.7/8.6 | 21.5/13.6 | 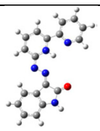 | *E-1-t1* |
| *Z-1-t1* | 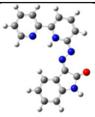 | 40.0 | *31.2* | 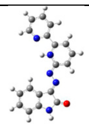 | *E-1-t2* |
| *Z-1-t2* | 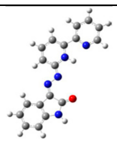 | 45.3 | *55.1* | 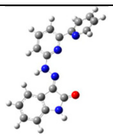 | *E-1-c1* |
| | | | *74.1* | 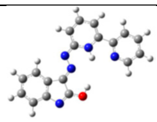 | *E-1-t3* |
| *Z-1-t3* | 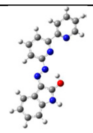 | 57.9 | *78.0* | 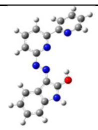 | *E-1-t4* |
| *Z-1-t4* | 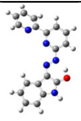 | 69.2 | *83.4* | 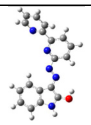 | *E-1-t5* |

| | 3 | | | 3 | |
|---|---|---|---|---|---|
| *Z-3* | 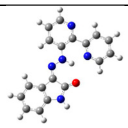 | 0/0 | 14.8/3.4 | 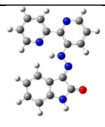 | *E-3* |
| *Z-3-c1* | 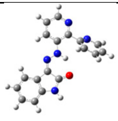 | 34.7/18.7 | 57.4 | 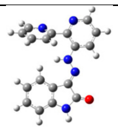 | *E-3-c1* |
| *Z-3-t1* | 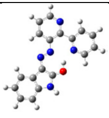 | 56.3 | 90.8 | 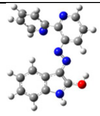 | *E-3-t1* |

| | | | | | | |
|---|---|---|---|---|---|---|
| ***Z-3-t*$_2$** | 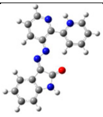 | **99.9** | | **96.9** | 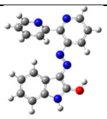 | ***E-3-t*$_2$** |
| **-** | - | **-** | | **113.7** | 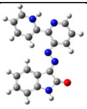 | ***E-3-t*$_3$** |
| | **4** | | | | **4** | |
| ***Z-4*** | 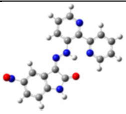 | **21.3** | | **0.0** | 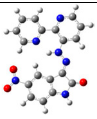 | ***E-4*** |
| ***Z-4-t*$_1$** | 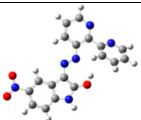 | **68.3** | | | | |
| ***Z-4-t*$_2$** | 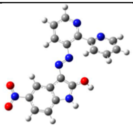 | **127.2** | | | | |

**Table S2.** Calculated Gibbs energies [M062x 6-31+g(dp)] level (T = 298.15 K) for *E* and *Z* isomers anions of studied hydrazones.

| *Anion Z$_{isomer}$* | **Compound** | **$\Delta G/\Delta G_{DMF}$ *kJ.mol$^{-1}$*** | | **$\Delta G/\Delta G_{DMF}$ *kJ.mol$^{-1}$*** | **Compound** | *Anion E$_{isomer}$* |
|---|---|---|---|---|---|---|
| | 3 | | | | 3 | |
| ***Z-3-A*$_1$*i*** | 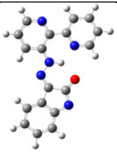 | **3.6/5.0** | | **0.0/0.0** | 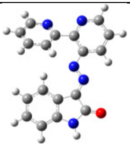 | ***E-3-A*$_1$*h*** |
| ***Z-3-A*$_1$*h*** | 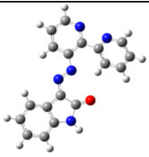 | **14.4/21.5** | | **5.6/39.5** | 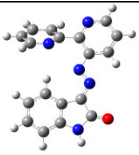 | ***E-3-A*$_2$*h*** |
| ***Z-3-A*$_2$*h*** | 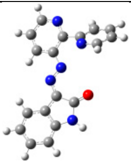 | **31,4/16.1** | | **34.7/15.9** | 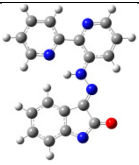 | ***E-3-A*$_1$*i*** |
